# What Do We Have to Know about PD-L1 Expression in Prostate Cancer? A Systematic Literature Review. Part 7: PD-L1 Expression in Liquid Biopsy

**DOI:** 10.3390/jpm11121312

**Published:** 2021-12-06

**Authors:** Andrea Palicelli, Martina Bonacini, Stefania Croci, Alessandra Bisagni, Eleonora Zanetti, Dario De Biase, Francesca Sanguedolce, Moira Ragazzi, Magda Zanelli, Alcides Chaux, Sofia Cañete-Portillo, Maria Paola Bonasoni, Stefano Ascani, Antonio De Leo, Jatin Gandhi, Alessandro Tafuni, Beatrice Melli

**Affiliations:** 1 Pathology Unit, Azienda USL-IRCCS di Reggio Emilia, 42123 Reggio Emilia, Italy; Alessandra.Bisagni@ausl.re.it (A.B.); Eleonora.Zanetti@ausl.re.it (E.Z.); Moira.Ragazzi@ausl.re.it (M.R.); Magda.Zanelli@ausl.re.it (M.Z.); mariapaola.bonasoni@ausl.re.it (M.P.B.); 2 Clinical Immunology, Allergy and Advanced Biotechnologies Unit, Azienda USL-IRCCS di Reggio Emilia, 42123 Reggio Emilia, Italy; Martina.Bonacini@ausl.re.it (M.B.); Stefania.Croci@ausl.re.it (S.C.); 3 Department of Pharmacy and Biotechnology (FABIT), University of Bologna, 40126 Bologna, Italy; dario.debiase@unibo.it; 4 Pathology Unit, Policlinico Riuniti, University of Foggia, 71122 Foggia, Italy; francesca.sanguedolce@unifg.it; 5 Department of Scientific Research, School of Postgraduate Studies, Norte University, Asunción 1614, Paraguay; alcideschaux@uninorte.edu.py; 6 Department of Pathology, University of Alabama at Birmingham, Birmingham, AL 35294, USA; scaneteportillo@uabmc.edu; 7 Pathology Unit, Azienda Ospedaliera Santa Maria di Terni, University of Perugia, 05100 Terni, Italy; s.ascani@aospterni.it; 8 Haematopathology Unit, CREO, Azienda Ospedaliera di Perugia, University of Perugia, 06129 Perugia, Italy; 9 Molecular Diagnostic Unit, Azienda USL Bologna, Department of Experimental, Diagnostic and Specialty Medicine, University of Bologna, 40138 Bologna, Italy; antonio.deleo@unibo.it; 10 Department of Pathology and Laboratory Medicine, University of Washington, Seattle, WA 98195, USA; jgandhi@uw.edu; 11 Pathology Unit, Department of Medicine and Surgery, University of Parma, 43121 Parma, Italy; alessandro.tafuni@unipr.it; 12 Fertility Center, Department of Obstetrics and Gynecology, Azienda USL-IRCCS di Reggio Emilia, 42123 Reggio Emilia, Italy; Beatrice.Melli@ausl.re.it; 13 Clinical and Experimental Medicine PhD Program, University of Modena and Reggio Emilia, 41121 Modena, Italy

**Keywords:** PD-L1, prostate, cancer, liquid biopsy, circulating tumor cells, exosomes, immunotherapy, checkpoint inhibitors

## Abstract

Liquid biopsy is an accessible, non-invasive diagnostic tool for advanced prostate cancer (PC) patients, potentially representing a real-time monitoring test for tumor evolution and response to treatment through the analysis of circulating tumor cells (CTCs) and exosomes. We performed a systematic literature review (PRISMA guidelines) to describe the current knowledge about PD-L1 expression in liquid biopsies of PC patients: 101/159 (64%) cases revealed a variable number of PD-L1+ CTCs. Outcome correlations should be investigated in larger series. Nuclear PD-L1 expression by CTCs was occasionally associated with worse prognosis. Treatment (abiraterone, enzalutamide, radiotherapy, checkpoint-inhibitors) influenced PD-L1+ CTC levels. Discordance in PD-L1 status was detected between primary vs. metastatic PC tissue biopsies and CTCs vs. corresponding tumor tissues. PD-L1 is also released by PC cells through soluble exosomes, which could inhibit the T cell function, causing immune evasion. PD-L1+ PC-CTC monitoring and genomic profiling may better characterize the ongoing aggressive PC forms compared to PD-L1 evaluation on primary tumor biopsies/prostatectomy specimens (sometimes sampled a long time before recurrence/progression). Myeloid-derived suppressor cells and dendritic cells (DCs), which may have immune-suppressive effects in tumor microenvironment, have been found in PC patients circulation, sometimes expressing PD-L1. Occasionally, their levels correlated to clinical outcome. Enzalutamide-progressing castration-resistant PC patients revealed increased PD-1+ T cells and circulating PD-L1/2+ DCs.

## 1. Introduction

Programmed cell death ligand 1 (PD-L1) is a 40 kDa transmembrane protein expressed by various activated immune cells (such as macrophages, dendritic, NK, or B cells) and different non-lymphoid tissues (including epithelial, endothelial, or muscle cells) [1,2]. It binds to its receptor PD-1, which is expressed by cytotoxic T, B, NK cells, and monocytes. The PD-1/PD-L1 interaction serves as an important regulatory checkpoint against an excessive adaptive immune response to antigens and autoimmunity, playing a key role in immune regulation and peripheral tolerance [1,2]. However, PD-L1 is also involved in the immune evasion process by tumor cells, including prostate cancer (PC) cells [1,2].

Novel biomarkers have been increasingly investigated to develop tailored therapies for various malignancies [3,4,5,6], and progressive attention has been paid to PD-1/PD-L1 checkpoint inhibitors. Moreover, the 2021 United States National Comprehensive Cancer Network (NCCN) guidelines have allowed the use of pembrolizumab (an anti-PD-1 monoclonal antibody) in selected PC patients [4]. 

Screening of PC is mainly based on prostate specific antigen (PSA) test, which unfortunately lacks sufficient diagnostic specificity and prognostic value. In particular, PSA has low efficiency in recognizing aggressive PCs, also resulting in a high percentage of overdiagnoses [4,7,8,9,10,11,12,13,14]. Early diagnosis of PC is a challenge for liquid biopsies, which may represent a non-invasive, real-time monitoring of tumor evolution and therapeutic efficacy through analysis of circulating elements, including tumor cells (CTCs), tumor DNA (ctDNA), tumor RNA (ctRNA), miRNAs, and extracellular vesicles [7,14]. 

In PC patients, PD-L1 has been demonstrated to be expressed by CTCs, also being identified in soluble exosomes in the bloodstream [7,14]. Monitoring PD-L1+ CTCs could reflect individual patient’s response to immunotherapy, representing a promising diagnostic and predictive aid [14]. Moreover, some evidence suggested that PD-L1 could be more expressed in advanced PCs, thus potentially identifying aggressive tumors [14]. Unfortunately, few data are available in literature as regards the PD-L1 expression in liquid biopsies of PC patients. In our systematic literature review, we tried to describe the current knowledge on this topic.

## 2. Materials and Methods

**Figure 1:**
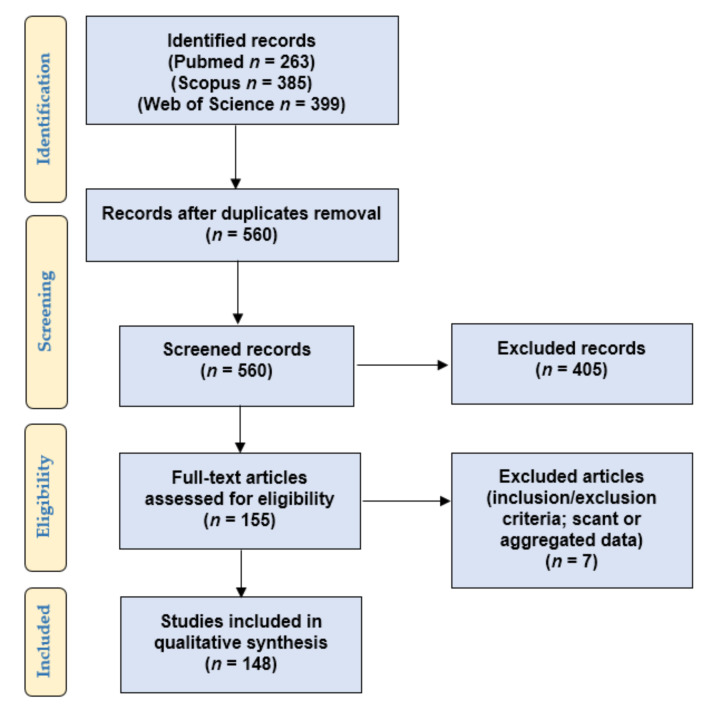
PRISMA flow-chart of the systematic literature review.

We followed the “Preferred Reporting Items for Systematic Reviews and Meta-Analyses” (PRISMA) guidelines (http://www.prisma-statement.org/; accessed on 8 May 2021) to conduct our systematic literature review about PD-L1 expression in PC (Figure 1).

Our retrospective observational study answered the following PICO (population, intervention, comparison, outcomes) questions:

- Population: patients and pre-clinical models (tumor cell lines, mouse models) included in studies investigating the role of PD-L1 in PC;
- Intervention: any treatment;
- Comparison: none;
- Outcomes: patient’s status at last follow-up (no evidence of disease, alive with disease, dead of disease), response to therapy, overall survival (OS), progression-free survival (PFS), biochemical recurrence-free survival (BCRFS), metastasis-free, cancer-specific, disease-free, or clinical failure-free survival. As regards experiments on PC cell lines and mouse models: any reported effect on cancer and immune cell migration, proliferation, viability, growth, resistance/response to therapy, cytotoxic/anti-tumor activity, PD-L1 expression, and mice/cell lines survival.

Eligibility/inclusion criteria: clinic-pathologic series (human patients) or experimental research (tumor cell lines, mouse models) concerning the role PD-L1 in PCs.

Exclusion criteria: non-primary prostatic cancers, non-carcinomatous histotypes, studies not examining PD-L1, review articles without cases, and cases of uncertain diagnosis.

We searched for (PD-L1 AND (prostate OR prostatic) AND (adenocarcinoma OR adenocarcinomas OR cancer)) in Web of Science (Topic/Title; 399 results; https://login.webofknowledge.com, accessed on 8 May 2021), Pubmed (all fields; 263 results; https://pubmed.ncbi.nlm.nih.gov, accessed on 8 May 2021), and Scopus (Title/Abstract/Keywords; 385 results; https://www.scopus.com/home.uri, accessed on 8 May 2021) databases. No limitations or additional filters were set. The bibliographic research ended on 8 May 2021.

To verify the satisfaction of the eligibility/inclusion criteria, two independent reviewers screened the titles and abstracts of the 560 records retrieved after excluding duplicate results. One hundred fifty-five eligible articles were retrieved in full-text format, and they were read by two other authors to look for additional relevant references and verify the inclusion and exclusion criteria. Seven papers were excluded for being unfit according to the inclusion criteria or for presenting scant or aggregated data. Two other authors checked the extracted data, and 148 articles were finally included in our study [8,9,10,11,12,13,14,15,16,17,18,19,20,21,22,23,24,25,26,27,28,29,30,31,32,33,34,35,36,37,38,39,40,41,42,43,44,45,46,47,48,49,50,51,52,53,54,55,56,57,58,59,60,61,62,63,64,65,66,67,68,69,70,71,72,73,74,75,76,77,78,79,80,81,82,83,84,85,86,87,88,89,90,91,92,93,94,95,96,97,98,99,100,101,102,103,104,105,106,107,108,109,110,111,112,113,114,115,116,117,118,119,120,121,122,123,124,125,126,127,128,129,130,131,132,133,134,135,136,137,138,139,140,141,142,143,144,145,146,147,148,149,150,151,152,153,154,155].

Data collection was study related (authors and year of study publication) and case related (tumor stage at presentation, Grade Group, type of specimen, treatment, test methods, results of PD-L1 expression, follow-up and outcomes, experiment type). 

The collected data were reported as continuous (analyzed by ranges, mean, and/or median values) or categorical variables (summarized by frequencies and percentages) for statistical analysis.

As there were many data to present, the discussion of our results was divided into different articles, focusing on distinct sub-topics. Here, we analyze the studies of PC patients investigating PD-L1 expression in liquid biopsies. 

## 3. Results

### 3.1. PD-L1 Expression in Blood Samples: An Overview

Few articles evaluated PD-L1 expression by tumor and/or inflammatory cells in blood samples of PC patients [11,14,16,20,23,45,54,64,87,88,91,96,97,101,147].

Globally, 613 cases were tested using different techniques (real-time polymerase chain reaction, RT-PCR; flow cytometry, FC; fluorescent in-situ hybridization, FISH).

The performed studies enrolled PC patients with or without a comparison with healthy donors. Clinic-pathologic features were rarely reported. When described, PCs were of various stage and Gleason score/Grade Group, and patients were variably treated. Correlation with clinical outcome was rarely investigated, sometimes revealing conflicting results.

### 3.2. PD-L1 Expression in Circulating Tumor Cells

When this information was reported, CTCs were found in all the tested PC cases, but selection biases were present [14,16,54,76,91,147]. Conversely, they were not found in the tested healthy donors. A total of 101/159 (64%) PC patients revealed PD-L1+ CTCs despite the range of CTCs and of PD-L1+ CTCs varied among patients; unfortunately, these data were not clearly reported in some series [14,16,54,76,91,147]. Two articles specified that PD-L1 positivity was found in >50% of CTCs in 31/60 (52%) cases [16,91]; however, in one of the two series, PD-L1 positivity was reported in the nuclei of CTCs (23/30, 77%), while in the second paper (as in the other reported studies), the PD-L1 staining seemed membranous. While no clear study identified correlations of PD-L1 expression by CTCs with OS, two researches found an association with PFS [91,147]. However, these correlations are still controversial: further studies are required. Here, we describe some details of the few published articles (Table 1).

Schott et al. [76] investigated the frequency of PD-L1 expression by CTCs of different cancers (prostate, *n* = 27; colorectal, *n* = 17; lung, *n* = 9; breast, *n* = 68) and in 25 healthy men (Extended Maintrac^®^ approach; FISH). This series included three stage I, four stage II, four stage III, and 12 stage IV PCs (unavailable data in four cases); eight PCs were pN1 (nine pN0, 10 pNx), while 15 cases showed distant metastases (11 M0, 1 Mx). Six patients were under 60 years of age, while 21 cases were >60 years. Six men underwent chemotherapy, while radiotherapy was administered to six patients. As all the 27 tested PC cases showed PD-L1+ CTCs (range: 32–100% of positive cells; median value: 65.8%), no clinic-pathologic correlation was possible among PC patients. A lower percentage of PD-L1+ CTCs was found in breast (94.5%), colorectal (94.5%), and lung (82%) cancer patients, while none of the healthy donors revealed CTCs [76]. Conversely, no PD-L1+ CTCs were found in another series of PC patients (*n* = 10) [54].

Treatment with DNA vaccination encoding prostatic acid phosphatase (PAP) increased PD-L1 expression on CTCs of PC patients; in the study of Rekoske et al. [147], this up-regulation correlated to the development of sustained antigen-specific IFN-γ-producing T cell immune response and to longer PFS. Multi-parameter FC was tested on cryopreserved peripheral blood mononuclear cells (PBMC) collected from men with PC at different stages and variably treated, including: group 1, non-castrate, non-metastatic, PSA-recurrent cases; group 2, castration-resistant, non-metastatic PSA-recurrent tumors; and group 3, metastatic, castration-resistant PCs (mCRPCs). The frequency of CD45-/EpCAM (Epithelial Cell Adhesion Molecule)+ CTCs was higher in castration-resistant patients; these cells also resulted positive for androgen receptor (AR), PAP, and CD63 (imaging cytometry), confirming the prostatic origin.

In the series of Satelli et al. [91], CTCs of 30 metastatic PCs (range: 1–890 CTCs) and 62 metastatic colorectal carcinomas (range: 1–20 CTCs) were tested by using magnetic separation with cell surface vimentin-specific monoclonal antibody (marker of epithelial-mesenchymal transition); nuclear PD-L1 expression was found in ≥50% of CTCs in 23/30 (77%) cases, being significantly associated with worse PFS (*p* = 0.0215) but not with worse OS (*p* = 0.0990), while CTC detection by itself did not correlate to unfavorable clinical outcome. 

Zhang et al. [16] enrolled 30 PC patients to test PD-L1 expression on their CTCs. The authors included 10 men with newly-diagnosed metastatic hormone-sensitive PCs (mHSPC) starting androgen deprivation therapy (group 1), 10 cases with mCRPCs starting abiraterone (ABT)/prednisone or enzalutamide (ENZ) (group 2), and 10 patients having mCRPCs progressing on ABT/prednisone or ENZ (group 3). The number of detectable CTCs differed between men, groups, and time. At baseline, all men had detectable CTCs, while 9/10 (90%) group 1, 6/10 (60%) group 2, and 4/10 (40%) group 3 patients revealed CTCs in their blood samples after 12 weeks. Moreover, only 2/10 (20%) group 1, 7/10 (70%) group 2, and 6/10 (60%) group 3 progressing patients showed detectable CTCs. In addition, not all the four tested samples collected at the same timepoint from each patient resulted positive. More than one PD-L1+ CTC was detected at baseline in 40% of group 1, 60% of group 2, and 70% of group 3 men, while >50% of PD-L1+ CTCs were found in 30% (group 1), 20% (group 2), and 30% (group 3) of the cases, respectively. PD-L2+ CTCs were found in 20–40% of patients in each disease stage, while CTLA-4 expression was rare (1–20%), and B7-H3 positivity rate was higher (80–90%). Among all cohorts, 27%, 23%, 10%, and 83% of men showed > 50% of CTCs positive for PD-L1, PD-L2, CTLA-4, and B7-H3, respectively. Men with mHSPC had more PD-L1+ CTCs over time, while the overall percentage of PD-L1+ CTCs decreased in the other two groups [16].

Zavridou et al. [14] (*n* = 62 mCRPCs) found a significantly higher positivity of gene expression markers (CK-8, CK-18, TWIST1, PSMA, AR-FL, AR-V7, AR-567, and PD-L1 mRNA) in EpCAM+ CTCs (PD-L1: 34/62, 54.8%) compared to plasma-derived exosomes (PD-L1: 15/62, 24.2%). Despite that it was not clearly stated, PD-L1 expression seemed not to correlate with OS.

### 3.3. PD-L1 Expression: Circulating Tumor RNA and Exosomes

Table 2 summarizes the few articles reporting some information about the role of ctRNA or exosomes in PD-L1 expression among PC patients [11,14,23,64].

Despite that ctRNA is more unstable than ctDNA in biological fluids (probably because of ribonucleases), some authors managed to isolate ctRNA of various cancer patients (prostatic, colic, gastric, and pulmonary carcinomas) by using RT-PCR [64]. Indeed, Ishiba et al. found ctRNA in the plasma of 21/88 (24%) PC patients by RT-PCR, while no PD-L1 mRNA was detected in cancer-free men (0/19, 0%); outcome correlation was not performed [64].

Zavridou et al. [14] reported that mCRPC patients expressed PD-L1 at lower levels in plasma-derived exosomes (15/62, 24.2%) than in EpCAM+ CTCs (34/62, 54.8%); although it was not clearly stated, the PD-L1 status seemed not to correlate with OS.

Vardaki et al. [11] found that plasma exosomes of patients with shorter OS had higher exosomal levels of PD-L1 at baseline compared to men with favorable prognosis; these changes were Radium-223 dependent, as there were no differences in the same immune checkpoint modulators upon cabazitaxel treatment. Immunohistochemical analysis on a tumor biopsy of a patient with unfavorable outcome revealed an agreement in PD-L1 expression between PD-L1 exosomal levels and immunohistochemical results. 

Immune checkpoints-related proteins (ICKRPs) and other soluble T cell regulatory factors released from immune and tumor cells may affect the efficacy of immunotherapy [23]. Wang et al. [23] evaluated serum levels of 14 ICKRPs (including PD-L1) and their potential correlation to clinical outcomes in a large series of 190 patients with localized PCs. Unlike soluble sPD-L1, sPD-L2 was significantly associated with BCRFS and PC progression (*p* < 0.05) as other soluble factors (such as sCD28, sCD80, sCTLA4, sHVEM, sIDO, sGITR, sPDCD1). The genotype profile of 97 single-nucleotide polymorphisms (SNPs) from 19 ICK-related genes was analyzed in an extended cohort of PC patients (*n* = 1762); the *CD274* gene (encoding for PD-L1) was correlated with biochemical recurrence and tumor progression. In particular, the SNP *CD274*:rs822335 showed the strongest association with PC progression among 22 SNPs (hazard ratio, 1.73, 95% confidence interval 1.31–2.29, *p* = 9.53E−05, *q*-value = 0.009). To validate whether the ICK-related genes were transcriptionally altered in PC, the authors retrieved the expression data of 19 ICK-related genes from The Cancer Genome Atlas Database (*n* = 494); *CD28, CD274, CTLA4, LAG3, PDCD1LG2, TNFRSF14, TNFRSF18,* and *TNFSF18* gene expression analysis revealed significant differences between tumor and normal tissues (*p* < 0.05). High levels of sBTLA and sTIM3 correlated with the risk of aggressive PC [23].

### 3.4. Studies on Circulating Immune Cells

Few studies provided some information about PD-L1 expression and data concerning circulating immune cells (Table 3) [20,45,54,87,88,96,97,101].

PD-L1 can also be expressed by circulating and intratumoral immune cells, including T (CD4+, CD8+), B, NK, dendritic, and myeloid-derived suppressor cells [54,156].

Tumor-associated stromal myofibroblasts or myeloid cells, such as macrophages (TAMs) or myeloid-derived suppressor cells (MDSCs), are negative prognostic factors in some malignancies, favoring tumor progression and metastases. Tumor-associated stroma may represent an immunosuppressive barrier to anticancer immunity, negatively influencing PC progression. Stromal-derived factors (such as IL-6) could favor the migration of myeloid cells, altering their differentiation into fully functional dendritic cells (DCs) and upregulating PD-L1; thus, PD-L1+ myeloid cells can suppress T cells in the tumor microenvironment [97]. Indeed, DCs acquire an immunosuppressive phenotype (CD14+/CD16+/CD68+/CD124+/CD209+; PD-L1 overexpression), becoming incapable of cross-presenting tumor antigens to T cells and inhibiting the T cell response [97]. Using FC, Spary et al. [97] performed a comparative phenotypic analysis between CD14+ tumor-infiltrating lymphocytes isolated from PC biopsies and CD14+ PBMCs of healthy donors. CD14+ tumor-infiltrating cells expressed higher levels of PD-L1 and CD209 than circulating T cells, while a higher percentage of tumor-associated CD3+ T cells expressed PD-1. CD209 is a C-type lectin receptor, which is present on the surface of macrophages and DCs; it activates the macrophagic phagocytosis by binding with high affinity to high-mannose type N-glycans (a class of pathogen-associated molecular patterns commonly found on viruses, bacteria, and fungi). On myeloid and pre-plasmacytoid DCs, CD209 mediates the DC interaction with blood endothelium, CD4+ T cell activation, and haptens recognition [97].

Stress and inflammation can trigger and/or sustain STAT3 activity in PCs, especially in immunosuppressive tumor-associated myeloid cells (macrophages, MDSCs). Cell death releases Toll-like receptor 9 (TLR9) ligands (such as mitochondrial DNA) in extracellular space and blood circulation; the TLR9/NF-kB-induced IL-6 secretion activates STAT3. High TLR9 expression and STAT3 activation in immunosuppressive polymorphonuclear MDSCs (PMN-MDSC; the major myeloid suppressor population) accumulate in the circulation of patients with PCs progressing from localized to metastatic/castration-resistant tumors. In PC models, STAT3 activity in tumor-infiltrating MDSCs correlated to increased PD-L1 levels and elevated plasma levels of IL6-type cytokines, such as LIF, suggesting a potential cross-talk mechanism promoting tumor immune evasion [157]. 

Sharma et al. reported that both CD14+ monocytic and CD14– granulocytic MDSCs expressed PD-L1 and PD-L2 (~2-fold greater in the latter type of cells); granulocytic MDSCs may suppress tumor-reactive CD8+ T cells in metastatic pelvic lymph nodes [88]. 

By using FC analysis, Zhou et al. [87] found a significant increase of MDSCs (*p* < 0.01) as well as of Arg-1, iNOS, and PD-L1 levels in the peripheral blood of 32 PC patients compared to 25 healthy controls. The distribution of CD14+ monocytic MDSCs and CD15+ PMN-MDSCs subsets significantly differed between the two groups (60.4% vs. 72.2%, 29.5% vs. 18.8%, respectively (*p* < 0.05). The percentages of MDSCs and monocytic MDSCs were remarkably associated with the survival rate (*p* = 0.025 vs. *p* = 0.017). 

ABT and ENZ were approved by the Food and Drug Administration (FDA) for the treatment of newly diagnosed metastatic androgen-sensitive PCs [158]. ABT inhibits the CYP17A1 enzyme involved in the synthesis of androgens in various body sites (adrenal glands, testes, etc.), as in PCs [158]. On the other hand, ENZ directly binds to the AR, preventing nuclear translocation and recruitment of the ligand-receptor complex [159]. Consensus regarding the optimal administration sequence in mCRPC patients is scant. De-novo ABT/ENZ resistance in mCRPCs can be related to the immunosuppressive tumor microenvironment. In the study of Pal et al. [45], ABT/ENZ therapy did not reduce the percentage of circulating tolerogenic myeloid cell populations, such as PMN-MDSCs in mCRPC patients. A total of 10% of 44 mCRPCs cases expressed PD-L1 on circulating PMN-MDSCs during ABT/ENZ treatment, also retaining unaltered B7-H3 expression. No difference in PD-1 expression among T cells was reported. 

Moreover, Bishop et al. found that progression on ENZ in CRPC patients was associated with increased frequency of PD-1+ T cells and circulating PD-L1/2+ DCs when compared to ENZ responders (*p* = 0.006) or naïve cases (prior to the start of treatment) (*p* = 0.0037) [96]. These findings increased with time, being associated with poor initial response to ENZ. Early ENZ responders with a <50% decrease in PSA had higher levels of circulating PD-L1/2+ DCs (vs. men showing >50% PSA decline after starting treatment). PD-L1/2+ DCs significantly increased with time to ENZ progression (*p* = 0.0497). PD-1+ T cells (CD4+ or CD8+) were increased without differences among patient subsets. Comparatively low expression of CTLA-4 on T cells was found across all patients. Pre-clinical results also reported significantly increased circulating PD-L1/2+ DCs in mice bearing ENZ-resistant tumors [96].

Clinical studies reported that administration of anti-CTLA-4 antibodies plus peptide vaccines resulted in a higher frequency of Th17 cells, improving survival [101,160]. Dulos et al. found that PD-1/PD-L1 blockade (not PD-L2) enhanced Staphylococcus Enterotoxin B-induced IL-2 production in healthy donors, shifting the antigen-induced cellular reactivity toward a pro-inflammatory Th1/Th17 response; it favored the production of IFN-γ, IL-2, TNF-α, IL-6, and IL-17, at the same time inhibiting the secretion of Th2 cytokines (IL-5, IL-13) [101]. This PD-1 blockade-induced shift toward a pro-inflammatory Th1/Th17 response detected in the peripheral blood may favor an antitumor cytotoxic T cell response in the tumor microenvironment [101,161]. Indeed, Foxp3+ regulatory T cells (Tregs) and Th17 differentiation are reciprocally regulated; in the study of Dulos et al., PD-1 blockade may either have caused Tregs inhibition or Th17 shift [101]. 

In-vitro binding studies using PBMCs of healthy donors showed that the anti-PD-L1 clone MIH1 can be used to detect PD-L1 after durvalumab exposure [54].

Finally, PD-L1 expression seemed increased on monocytes/DCs after short-course radiotherapy (*p* = 0.047; *p* = 0.031) [20].

## 4. Discussion

While tissue biopsies are invasive and sometimes technically challenging, liquid (blood) biopsy may be an easily accessible, non-invasive diagnostic tool for advanced cancer patients (including men with PC), potentially representing a real-time monitoring test for tumor evolution and response to treatment through analysis of CTCs, ctDNA, ctRNA, circulating miRNAs, and exosomes. Liquid biopsies minimize the risk for patients, being especially helpful when metastatic sites are unreachable by tissue biopsies [76,87,88,91,96,97,101,147,148].

CTCs are accessible precursors of metastatic disease, being detectable like other blood cells. CTCs may be more frequent in men with advanced PCs, and their presence seems to parallel tumor burden, aggressiveness, and response to therapy. However, CTCs may also be identified in localized, early-stage PC cases, with a detection rate ranging from 5% to 52%. Although thousands of tumor cells can be released into the blood and/or lymphatic circulation by aggressive PCs, only <0.01% of CTCs eventually succeed in forming metastases. Moreover, CTCs could be cleared from the blood before reaching detectable levels. Finally, localized cancers may release an insufficient number of CTCs, decreasing the sensitivity of liquid biopsies. To our review, the range of CTCs widely varied among patients, and in some cases, just one CTC was identified [14,16,54,76,91,147,162,163,164,165]. 

When CTCs are scant, their identification represents a technical challenge. RT-PCR has high sensitivity and specificity, detecting one prostatic cell among 10^6^–10^8^ hematopoietic cells in peripheral blood [166]. Different methods of epithelial cells production/enrichment may extract CTCs from peripheral blood. Sample handling is fundamental to obtain reliable and reproducible results in terms of applicability, specificity, and clinical impact. Expression of surface or intracellular antigens can immunologically discriminate circulating cells usually absent in healthy patients [167]. Laser scanning cytometry is fast, as is FC, and can analyze every single positive event for its morphological properties: in 30 min, it may quantify up to 50,000 live tumor cells through exclusive surface staining, omitting dead cells with intracellular staining and discriminating between non-specific fluorescence events and true cells [167].

The EpCAM-based immunocapture technique is frequently used for isolating CTCs. The FDA approved the CellSearch system (Menarini, Italy) for counting CTCs in the peripheral blood of mCRPC patients in clinical practice; CTCs have been validated as a prognostic biomarker of PCs in different clinical trials [14,168,169,170]. This semi-automated platform enriches CTCs based on EpCAM and cytokeratin (CK8, CK18, CK19) expression and on CD45-negativity [14,168,169,170]. In a study [170], favorable CTC counts (<5 cells/7.5 mL blood) predicted significantly improved PFS and OS in ABT-treated PC patients; conversion of favorable CTC counts to unfavorable values (and vice versa) were associated with parallel prognostic improvement/deterioration. The CTC count was considered as an early response marker (detectable 2–5 weeks after starting the treatment), outperforming a 30–50% decline in PSA levels (prognostically significant after 6–8 weeks). Unfortunately, other authors found that this technique failed to isolate EpCAM+ CTCs in ~26.2% of CRPC patients [132]. The false negativity may be due to an epithelial-mesenchymal transition (EMT) of tumor cells during progression; like in tissue specimens, CTCs may downregulate the epithelial markers (such as EpCAM), upregulating mesenchymal proteins [14]. The EpicScience platform (Epic Science, USA) uses high-throughput imaging (fiber-optic array scanning technology) to identify the CTCs and leukocytes labeled with cytokeratins, CD45, and/or 4′6-diamidino-2-phenylindole (DAPI); it is an EpCAM expression-independent technique [171]. The CK-negative CTCs may be more aggressive, as are those showing neuroendocrine differentiation; there is a need for assays also based on new markers [172].

Other techniques have been proposed to capture CTCs, such as those based on the expression of *MET* oncogene [173]. To detect CTC-associated transcripts, some authors analyzed the mRNAs derived from whole blood, without prior CTC enrichment [174,175]. Other platforms (Adnatest^®^, Qiagen GmbH, Germany) combined immunomagnetic enrichment of CTCs with RNA isolation and RT-PCR [176,177]. Magnetic separation with a cell-surface vimentin-specific 84-1 monoclonal antibody may detect CTCs with EMT, predicting response to treatment in PC patients [91]. Label-free methods based on cell size and morphology could separate CTCs from the blood by using three-dimensional microfilters and bilayers [178,179,180,181]. However, pore size and rigidity of the membrane could affect the success rate. Moreover, a high flow could squeeze CTCs through pores, while leukocytes accumulation and blood clotting can be induced by slow flow rates [178,179]. In some studies, microfluidic devices resulted in higher CTC counts [180,181]. Multiple-antibodies-functionalized microfluidic devices may isolate different CTC-subpopulations [131]. 

If non-dissipative methods are used (avoiding cell loss), the CTC count changes represent a good marker for response to treatment, allowing continuous real-time monitoring during therapy. Cancer-specific biomarkers (especially if predictive of more aggressive tumors) are increasingly investigated in combination with the CTC count [167,182].

CTCs may reflect alterations in the tumor microenvironment better than archival tissue specimens, helping the monitoring of cell surface changes [183]. The PD-1/PD-L1 pathway is involved in tumor immune escape, favoring disease progression. PD-L1 has been identified on CTCs in metastatic breast, prostate, colorectal, lung, and urothelial cancers [16]. Immune-checkpoint ligands (PD-L1, PD-L2, CTLA-4) are heterogeneously expressed on PC-CTCs among different disease settings, cohorts of patients, and timepoints [16]. 

PD-L1 immunohistochemical (IHC) expression could occur more frequently in high-risk localized or metastatic PC tissues, potentially correlating to more aggressive clinic-pathologic features and outcome despite some limits and controversial results (as described in other parts of our review) [27,35,36,39,43]. PD-L1+ PCs may be more commonly associated with lymph node metastases [39] or higher risk for developing distant metastases [77]. The high percentage of PD-L1+ CTCs found in some series may be in keeping with the hypothesis that the more aggressive PCs are PD-L1+ and that CTCs may better reflect the current status of the disease. In particular, all the 27 PC patients of Schott et al. revealed PD-L1+ CTCs in blood. Possible explanations include the easier accessibility of surface antigens in CTCs and/or the fact that, in the bloodstream, CTCs are continuously in contact with T lymphocytes, which can favor PD-L1 expression by direct contact or cytokine secretion [76]. Indeed, after tumor antigen recognition, the T cell-induced IFN-γ signals favor antitumor effects (increased antigen presentation, chemokines, tumor growth arrest, and apoptosis), but they also cause an adaptive increase in PD-L1 expression on the tumor cells, allowing their escape from cytotoxic T cells [184]. However, PD-L1+ CTCs may also be found in patients without clinically-confirmed metastases; further studies need to verify the impact of these data on treatment decisions [76].

A promising correlation between OS and the CTC count was suggested for various metastatic tumors [185]. In PC patients, some authors found an association of PD-L1 expression by CTCs with PFS, while no clear correlation with OS was identified [91,147]. However, the published series usually represented monocentric studies, frequently showing selection biases and testing few patients [14,16,54,76,91,147]; independent validation multicenter cohorts are required. Moreover, correlation between soluble and cellular immune-checkpoint-related proteins in peripheral blood is largely unknown.

PD-L1 could be aberrantly expressed by various tumors, being mislocalized into the nucleus. Nuclear PD-L1 expression (nPD-L1) seems to promote drug resistance, indicating poor prognosis (such as cancer progression and metastasis). Indeed, Satelli et al. found that PC-CTC detection by itself did not correlate to poor PFS or OS in PC patients. Conversely, nuclear (not membranous) PD-L1 expression was significantly associated with shorter PFS in PC patients although few men were tested [91]. However, in another study (*n* = 171) [12], tumor stage was significantly higher in nPD-L1+ PCs, but neither nuclear nor membranous PD-L1 positivity were predictive of BCRFS on univariate and multivariate analyses [12]. Chemotherapy may induce nuclear translocation of PD-L1, suggesting that this marker has functions other than T cell inhibition [186,187].

PD-L1+ CTCs may help to select patients for treatment with checkpoint inhibitors [76]. PD-L1 IHC expression alone may be insufficient to predict the clinical response to immunotherapy. Indeed, the heterogeneous, usually focal, PD-L1 staining in PC tissue samples questions the reliability of IHC analysis alone as a predictor of clinical outcome and response to therapy [8,9,10,11,12,13,14,15,16,17,18,19,20,21,22,23,24,25,26,27,28,29,30,31,32,33,34,35,36,37,38,39,40,41,42,43,44,45,46,47,48,49,50,51,52,53,54,55,56,57,58,59,60,61,62,63,64,65,66,67,68,69,70,71,72,73,74,75,76,77,78,79,80,81,82,83,84,85,86,87,88,89,90,91,92,93,94,95,96,97,98,99,100,101,102,103,104,105,106,107,108,109,110,111,112,113,114,115,116,117,118,119,120,121,122,123,124,125,126,127,128,129,130,131,132,133,134,135,136,137,138,139,140,141,142,143,144,145,146,147,148,149,150,151,152,153,154,155]. Moreover, patients with absent or low (<1%) PD-L1 positivity could also benefit from immunotherapy [112,188].

The failure of the PD-1/PD-L1 pathway blockade in PC treatment could also be due, at least in part, to the lower expression of PD-L1 by tumor cells [94]. We found that 29% acinar PCs, 7% ductal PCs, and 46% neuroendocrine carcinomas/tumors were PD-L1+ by IHC despite the influence of pre-analytic variables. Different scoring criteria and antibody clones were used in the tested series (usually monocentric), frequently including limited or unselected cases with variable clinic-pathologic features (age, stage, Grade Group, etc.) [8,9,10,11,12,13,14,15,16,17,18,19,20,21,22,23,24,25,26,27,28,29,30,31,32,33,34,35,36,37,38,39,40,41,42,43,44,45,46,47,48,49,50,51,52,53,54,55,56,57,58,59,60,61,62,63,64,65,66,67,68,69,70,71,72,73,74,75,76,77,78,79,80,81,82,83,84,85,86,87,88,89,90,91,92,93,94,95,96,97,98,99,100,101,102,103,104,105,106,107,108,109,110,111,112,113,114,115,116,117,118,119,120,121,122,123,124,125,126,127,128,129,130,131,132,133,134,135,136,137,138,139,140,141,142,143,144,145,146,147,148,149,150,151,152,153,154,155]. Moreover, discrepancy in PD-L1 IHC expression between primary and metastatic sites is a documented reality in PC [74]. Furthermore, intratumoral heterogeneity of PD-L1 staining (usually focally expressed) in either primary or metastatic PCs, small sample size, and lack of standardization limit the interpretation especially of single-core biopsies. In addition, hypofixation (as in case of huge prostates) or overfixation of tumor tissue, as well as decalcification procedures (in samples derived from bone metastases), may alter or destroy surface antigens, such as PD-L1 [8,9,10,11,12,13,14,15,16,17,18,19,20,21,22,23,24,25,26,27,28,29,30,31,32,33,34,35,36,37,38,39,40,41,42,43,44,45,46,47,48,49,50,51,52,53,54,55,56,57,58,59,60,61,62,63,64,65,66,67,68,69,70,71,72,73,74,75,76,77,78,79,80,81,82,83,84,85,86,87,88,89,90,91,92,93,94,95,96,97,98,99,100,101,102,103,104,105,106,107,108,109,110,111,112,113,114,115,116,117,118,119,120,121,122,123,124,125,126,127,128,129,130,131,132,133,134,135,136,137,138,139,140,141,142,143,144,145,146,147,148,149,150,151,152,153,154,155,189]. These factors may lead to false-negative results, depriving patients of treatment benefits [76].

The primary tumor is a debatable choice for determining PD-L1 IHC expression in metastatic PC patients. Metastases may better represent the more clinically-relevant tumor clones that have avoided immune destruction, disseminating into the circulation [76]. Moreover, results of PD-L1 IHC analysis derived from biopsies or radical prostatectomy samples (collected years before treatment and/or the detection of metastases) may be less relevant compared to the characterization and dynamic serial monitoring of CTCs at the time of progression/recurrence, when immunotherapy could be indicated. Indeed, PD-L1 expression may change among the various tumor sites (primary vs. metastases) as well as over time [16]. 

Unfortunately, there are limited data concerning studies evaluating PC-CTCs and PD-L1 expression. In our review (when this information was available), the majority of PC patients revealed PD-L1+ CTCs; however, data were not always clear, and selection biases may have occurred. Moreover, the variation in the number of CTCs (sometimes <10 CTCs) and of PD-L1+ CTCs among patients represented a bias for their detection and for the evaluation of the PD-L1 positivity rate. In addition, there are no standardized scoring systems or cut-off criteria for PD-L1 expression by CTCs. In tissue-based studies, PD-L1 IHC expression was analyzed by using several scoring systems (tumor proportion score, combined positive score, etc.), which have found their way into the clinic as companion diagnostics for other tumors. Conversely, PD-L1 expression by CTCs was usually described as positive or negative, sometimes including nuclear positivity (typically not assessed in IHC tissue-based studies); the exact number of PD-L1+ CTCs and clear cut-offs or scoring systems to qualify a patient as “positive” were unclear or not established, as few cases were tested [14,16,54,76,91,147]. Furthermore, discordance in PD-L1 status was not only detected among different tumor tissue sites (primary vs. metastatic) but also between CTCs and tumor tissues of the same patients. Schott et al. included 128 cancers arising from prostate (*n* = 27), breast (*n* = 72), colon-rectum (*n* = 18), and lung (*n* = 11); the frequency of PD-L1+ patients and of PD-L1+ CTCs was higher than the PD-L1 expression rate of tumor tissues [76].

CTCs can be sampled and characterized at any time during the course of disease, providing important data on therapeutic targets and drug resistance mechanisms [76]. After immunotherapy administration, the total CTC number and the fraction of PD-L1+ CTCs were significantly reduced in a study [76]. The rate of PD-L1+ CTCs may continuously increase during discontinuous drug administration. However, the persistence of PD-L1+ CTCs apparently correlates with poor prognosis and resistance to therapy [190]. 

In some studies, the post-vaccination increase in PD-L1 expression by CTCs correlated to the development of sustained antigen(PAP)-specific IFN-γ-secreting T cell immunity and longer PFS [147,148]. Dynamic monitoring of CTCs (expressing PD-L1 or other biomarkers) could help in assessing antitumor immunity during various treatments, defining personalized vaccination schedules and/or selecting patients likely to have clinical benefit. Non-T, IFN-γ-secreting cells (including NK cells) may influence PD-L1 expression on CTCs [147,148].

Similar findings were reported in patients treated with Sipuleucel-T, an FDA-approved autologous cellular vaccine for targeting PAP antigen and improving OS in metastatic PCs. Early-stage PCs may have pre-existing PAP-specific delayed-type hypersensitivity responses regulated by CTLA-4. In various PC patients, Sipuleucel-T can induce different increases in Th1 cytokine secretion but similar (although not as strong) PD-L1 upregulation on CTCs following immunization. Sipuleucel-T may elicit mixed Th1/Th2 immune responses, producing antibodies and Th2 cytokines not observed with DNA immunization. Advanced PCs with greater tumor burden could also affect the rate of CD8+ T cells detected in the peripheral blood [147,148,191,192].

Liquid biopsy may be useful for a dynamic immunophenotypic or molecular characterization of CTCs (including PD-L1 expression) [76]. RT-PCR amplifies the signals, helping gene expression analysis; it could be used downstream to different molecular assays for studying CTCs and exosomes. CTCs show heterogeneous genetic differences from primary tumor cells, altering the molecules related to cell adhesion, migratory capacity, and angiogenesis [14]. Zavridou et al. suggested that the detection of AR-V7 or other specific markers in CTCs could help in selecting hormone therapy or chemotherapy in mCRPCs, while DNA methylation-based markers on CTCs and exosomes may provide relevant information on the epigenetic silencing of genes implied in the metastatic behavior [14,176,193,194]. CTC molecular characterization may identify new prognostic and predictive biomarkers; it could also favor the study of the biology of metastasis and the mechanisms of resistance to various drugs (ENZ, ABT, prednisone, and taxanes) [195,196,197]. Genomic profiling of PC-CTCs found that hormone therapy resistance can be due to AR mRNA splice variants, AR signaling loss, and/or gain of neuroendocrine-like features [16]. 

Soluble T cell regulatory immune checkpoint-related proteins released from immune and tumor cells may affect treatment and outcome efficacy [23]. Exosomes are extracellular vesicles secreted by normal and tumor cells in the extracellular space and blood circulation; they originate from fusion of multivesicular bodies with the plasma membrane, being surrounded by a lipid bilayer and favoring intercellular communication by transferring lipids, proteins, DNA, RNA, and metabolites to recipient cells [166,198,199,200]. ctDNA can also be released by dead (as to apoptosis, necrosis) or viable cells, also through exosomal vescicles. ctDNA levels ranges from 1 to 10 ng/mL in healthy donors, while cancer patients usually show increased ctDNA levels [166,198,199,200]. 

Smaller exosomes (diameter: 50–100 nm) may originate from benign or malignant cells, while large oncosomes (1–10 μm) seem to preferentially derive from tumor cells [166,198,199,200]. Differential ultracentrifugation is the gold standard for their detection, while ultrafiltration- and immunoaffinity-based approaches could also be beneficial. Selective biomarkers expressed by exosomes/oncosomes may improve their identification within the heterogeneous exosomal population; isolation techniques must be tailored to the specific vesicles of interest [166,198,199,200]. 

Exosomes are involved in cancer growth, neo-angiogenesis, development of pre-metastatic niches, resistance to treatments, tumor progression/recurrence, and immunosuppression, representing a valuable source of cancer biomarkers and being potentially involved in the metastatic progression of PC [14,166,198,199,200]. However, exosomes could also play an antitumor function in selected microenvironment contexts; DCs could activate B and T cells via exosomes (anti-tumor effect), while immunosuppression (Tregs activation, NK cells inhibition) could be favored by cancer cell-derived exosomes. Moreover, exosomal PD-L1 suppressed antitumor immunity in various tumor types, mediating resistance to immunotherapy by directly binding to anti-PD-L1 antibodies [166,198,199,200].

Zavridou et al. [14] reported that PD-L1 expression was higher in EpCAM+ CTC samples (34/62, 54.8%) than plasma-derived exosomes (15/62, 24.2%). However, this difference may be due to the variable sample amounts used for analysis: exosomes were isolated from 2 mL of plasma while CTCs from 20 mL of peripheral blood.

Two different tumor-secreted PD-L1 splicing variants were identified, lacking the transmembrane domain. Tumor cells could release extracellular exosomes carrying most of the PD-L1 produced upon IFN-γ stimulation (instead of transporting it to the cell surface), thus suppressing the T cell function and contributing to the resistance to PD-L1 blockade treatment [201]. Therefore, PD-L1 may not require a cell-to-cell interaction to inhibit T cell response and cause immune evasion; controlling exosomal PD-L1 levels might enhance the efficacy of anti-PD-L1 treatment in PC [202].

The tumor-associated metzincin metalloproteinases ADAM10 and ADAM17 can cleave the PD-L1 ectodomain from the surface of tumor cells and extracellular vesicles, thus producing a soluble form of PD-L1 (sPD-L1), which can induce CD8+ T cell apoptosis. This mechanism of resistance to PD-L1 or PD-1 inhibitors may explain why high serum sPD-L1 levels are predictors of poor response to immunotherapy and/or aggressive behavior in some malignancies; sPD-L1 may also act as a sink for circulating PD-L1 inhibitors. To this hypothesis, anti-PD-L1/PD-1 drugs may be administered to selected patients previously treated with plasma exchange to remove circulating sPD-L1. However, further studies are required, as high sPD-L1 levels are a positive prognostic indicator in a minority of tumor types (such as gastric adenocarcinoma) [116]. Unlike sPD-L2, sPD-L1 was not significantly associated with BCRFS and PC progression in a single study of PC patients, while the SNP *CD274:rs822335* showed the strongest association with PC progression in an extended cohort [23]. 

Systematic literature reviews allow clinicians to stay up to date, representing a starting point for developing clinical practice guidelines or further studies/trials and a justification for research financial support by granting agencies. Usually requiring a multidisciplinary approach and high effort by consolidated research teams, the PRISMA guidelines include an evidence-based minimum set of items for reporting and could be applicable in various topics/contexts, improving the quality of pure meta-analyses and case report/series [203,204,205,206,207,208,209,210,211,212,213,214,215,216,217,218,219,220,221,222,223,224,225,226,227,228,229,230,231,232,233,234,235,236,237,238,239,240,241,242,243,244]. We divided the presentation of our data into different papers, each focused on a specific sub-topic. In the other articles, the readers will find further information about PD-L1 IHC expression in PC patients (including discussion of pre-analytical and interpretation variables; correlation with clinic-pathologic features, the status of mismatch repair system, *BRCA*, *PTEN* and other main genes; results of clinical trials) and regulation of PD-L1 expression in pre-clinical PC models (intracellular signaling pathways, role in tumor microenvironment; experimental treatments on PC cell lines or mice; genetic and epigenetic regulation) [245,246,247,248,249].

## 5. Conclusions

Liquid biopsy is an accessible, non-invasive sampling technique and aid in metastatic PC patients. A variable number of CTCs expressed PD-L1 in 64% of the tested cases. Discordance in the PD-L1 status was detected between primary and metastatic PC tissue biopsies as well as between CTCs and the corresponding tumor tissues. Nuclear PD-L1 expression by CTCs was occasionally associated with worse prognosis. Outcome correlations should be investigated in larger series. 

PD-L1 could also be expressed by circulating immune-cells. MDSCs and DCs may have immune-suppressive effects in the tumor microenvironment; they have been found in the circulation of PC patients, sometimes expressing PD-L1. Occasionally, their levels correlated to the survival rates. ENZ-progressing castration-resistant PC patients revealed increased PD-1+ T cells and circulating PD-L1/2+ DCs.

PD-L1 is also released by PC cells through soluble exosomes, which could inhibit the T cell function and cause immune evasion.

Liquid biopsy potentially represents a real-time monitoring test for tumor evolution and response to immunotherapy through analysis of PD-L1+ CTCs and exosomes. Various treatments (ABT, ENZ, radiotherapy, checkpoint-inhibitors) may influence the PD-L1+ CTC levels. Genomic profiling of PD-L1+ PC-CTC could better characterize metastatic PCs compared to the evaluation of PD-L1 expression on primary tumor biopsies or prostatectomy specimens (sometimes sampled a long time before recurrence/progression). 

## Figures and Tables

**Table 1:** PD-L1 expression in circulating tumor cells.

Ref.	Test	Samples	Stage	Treatment	Results
[16]	CellSearch^®^ system (Menarini Silicon Biosystems, PA, US) (PD-L1 antibody clone SP142, Ventana Medical Systems, AZ, US)	30 PCs HD spiking experiments (unclear number)	NR	10 pre-ARSI 10 post-ARSI 10 mHSPC	Among all cohorts, the rate of men with >50% of CTCs positive for PD-L1 was 27%, for PD-L2 was 23%, for B7-H3 was 83%, and for CTLA-4 was 10% (*). At baseline, ≥1 PD-L1+ CTC was found in 40% of mHSPC (*n* = 4), 60% of mCRPC pre-ARSI (*n* = 6), and 70% of mCRPC post-ARSI (*n* = 7); ≥50% CTCs were PD-L1+ in 30% (*n* = 3), 20% (*n* = 2), and 30% (*n* = 3) of cases, respectively. Over time, mHSPCs released more PD-L1+ CTCs, while the overall percentage of PD-L1+ CTCs decreased in the other 2 groups.
[14]	qRT-PCR	62 mCRPCs 10 HDs	Var	62 prior ChT or new hormonal agents	Significantly higher positivity of gene expression markers (CK-8, CK-18, TWIST1, PSMA, AR-FL, AR-V7, AR-567, and PD-L1 mRNA) in EpCAM+ CTCs compared to plasma-derived exosomes (PD-L1: 34/62, 54.8% vs. 15/62, 24.2%).
[76]	Maintrac^®^ (PD-L1 clone 29E.2A3, Biolegend, CA, US) FISH (CD274/CEN9q probe, Abnova, Taiwan)	27 PCs 25 HDs	I–IV (N0/1, M0/1)	ChT (6); RT (6)	100% of PC patients and 0% of HDs showed PD-L1-positive CTCs (range 32–100% positive cells, median 65.8%). No correlation with clinic-pathologic parameters among PC patients.
[147]	FC (PD-L1-PECy7, clone MIH1, eBioscience, CA, US)	15? PCs	M0-1	unclear (some CRPCs)	Patients who developed long-term prostatic acid phosphatase-specific immune responses had PD-L1 upregulation on CTCs. Increased PD-L1 expression correlated to longer PFS.
[91]	FC (PD-L1 rabbit monoclonal, Invitrogen)	30 PCs	M1	ChT (palliative)	Nuclear PD-L1 expression in ≥50% of CTCs in 23/30 (77%) cases. It was significantly associated with worse PFS (HaR: 38.39; 95% CI, 1.714–859.700; *n* = 10; *p* = 0.0215) but not with OS (HaR 4.060; 95% CI, 0.7684–21.4600; *n* = 30; *p* = 0.0990). CTC detection alone was not associated with poor OS (HaR 1.182, 95% CI 0.2400–5.8230, *n* = 30, *p* = 0.8369) or PFS (HaR 0.2739, 95% CI 0.009854–7.614000, *n* = 10, *p* = 0.4452).
[54]	FC	10 PCs (°)	M1	Dur + Ola to mCRPCs (prior ENZ/ABT)	No patient showed PD-L1 positivity.

ABT, abiraterone; ChT, chemotherapy; CI, confidence interval; CRPC, castration-resistant prostate cancer; CTC, circulating epithelial tumor cells; Dur, durvalumab; ENZ, enzalutamide; FC, flow cytometry; HaR, hazard ratio; HD, healthy donors; mCRPC, metastatic castration-resistant prostate cancer; mHSPC, metastatic hormone-sensitive prostate cancer; NR, not reported; Ola, Olaparib; OS, overall survival; PC, prostate cancer; PFS, progression-free survival; post-ARSI, mCRPC progressing on ENZ or ABT/prednisone; pre-ARSI, mCRPC starting ENZ or ABT/prednisone; qRT-PCR, Quantitative Real-Time Polymerase Chain Reaction; Ref., references; RT, radiotherapy; Var, variable. Note: in all these cases, the Gleason Score/Grade Group of prostatic carcinoma was variable or unreported. (*): At baseline, at least 1 PD-L2+ CTC was found in 40% of mHSPCs, 40% of mCRPCs pre-ARSI, and 20% of mCRPCs post-ARSI; ≥50% CTCs were PD-L2+ in 30%, 20%, and 30% of cases, respectively. At baseline, at least 1 CTLA-4+ CTC was found in 10% of mHSPCs, 20% of mCRPCs pre-ARSI, and 10% of mCRPCs post-ARSI; ≥50% CTCs were CTLA-4+ in 10% of the cases of each group. At baseline, at least 1 B7-H3+ CTC was found in 90% of mHSPCs, 80% of mCRPCs pre-ARSI, and 90% of mCRPCs post-ARSI; ≥50% CTCs were B7-H3+ in 80%, 80%, and 90% of cases, respectively. (°): PD-L1 was also evaluated in 2/10 cases by immunohistochemistry. 12/17 (71%) cases of another cohort revealed CTCs at baseline (range: 0–2107), but it was unclear if these cases were tested for PD-L1 expression; the CTC count decreased or remained unchanged from treatment day 1/cycle 1 to day 15/cycle 1 in 13/17 (76%) cases, and these patients revealed better PFS.

**Table 2:** PD-L1 expression: circulating tumor RNA and exosomes.

Ref.	Test	Samples	Stage	GS	Treatment	Results
[64]	qRT-PCR	88 PCs 19 HDs	pM1	NR	NR (ChT?)	21/88 (24%) of PC patients and 0/19 (0%) of HDs showed PD-L1 expression in plasma circulating tumor RNA.
[14]	qRT-PCR	62 mCRPCs 10 HDs	Var	Var	prior ChT or new hormonal agents (*n* = 62)	Significantly higher positivity of gene expression markers (CK-8, CK-18, TWIST1, PSMA, AR-FL, AR-V7, AR-567, and PD-L1 mRNA) in EpCAM+ CTCs than plasma-derived exosomes (PD-L1: 34/62, 54.8% vs. 15/62, 24.2%).
[11]	WB, LMA	25 PCs	IV	Var	Var, including RT/Radium-223	Plasma exosomes of patients with unfavorable OS (*n* = 12) had higher levels of PD-L1 than men with favorable prognosis (*n* = 13) (Radium-223-dependent changes; no differences in the same immune checkpoint modulators upon cabazitaxel treatment).
[23]	FC (ProcartaPlex Human Immuno-Oncology Checkpoint Pane, Thermo Fisher, US sBTLA) (°)	190 PCs (95 LR, 95 HR)	LR (95 T1) HR (34 T1, 12 T2, 47 T3-4)	LR: 6 HR: 92% >6	84 RP; 41 RT; 60 Surv; 5 Cry	Serum PD-L1 levels did not correlate with BRFS (Har 4.8; 95% CI 0.9–25.7; *p* = 0.06; *q* = 0.084) or PFS (Har 4.0; 95% CI 0.8–20.9; *p* = 0.100; *q* = 0.140). Serum PD-L2 levels were significantly associated with BRFS (Har 5.5; 95% CI 1.2–26.2; *p* = 0.030; *q* = 0.053) and PFS (Har 4.6; 95% CI 1.1–18.5; *p* = 0.030; *q* = 0.047).

(°): this study evaluated the serum levels of 14 immune checkpoint markers (sCD27, sCD28, sCD80, sCD137, sCTLA4, sGITR, sHVEM, sIDO, sLAG3, sPDCD1, sPD-L1, sPD-L2, sTIM3). BRFS, biochemical recurrence-free survival; ChT, chemotherapy; CI, confidence interval; Cry, cryoablation; CTC, circulating epithelial tumor cells; FC, flow cytometry; GS, Gleason score; Har, hazard ratio; HD, healthy donors; HR, high-risk prostate cancer; LMA, Luminex multiplex array; LR, low-risk prostate cancer; mCRPC, metastatic castration-resistant prostate cancer; NR, not reported; OS, overall survival; PC, prostate cancer; PFS, progression-free survival; qRT-PCR, Quantitative Real-Time Polymerase Chain Reaction; Ref., reference; RP, radical prostatectomy; RT, radiotherapy; Surv, surveillance; Var, variable; WB, Western blot analysis.

**Table 3:** PD-L1 expression in circulating immune cells.

Ref.	Test	Samples	Stage	Treatment	Results
[20]	FC	14	LR, IR	RT (monotherapy) (*)	After 1 weak of treatment, short-course RT significantly increased PD-1 expression on T cells (*p* = 0.016), PD-L1 expression on monocytes (*p* = 0.047), and plasmacytoid DCs (*p* = 0.031) compared to pre-treatment and standard RT (*p* = 0.017, *p* = 0.026, and *p* = 0.035, respectively).
[45]	FC	44	M1	11 ABT, 23 ENZ, 5 ENZ + ABT, 7 ABT + ENZ	ABT/ENZ therapy did not affect the expression of PD-L1 and B7-H3 on circulating polymorphonuclear MDSCs in mCRPCs (10% expressed PD-L1 on polymorphonuclear MDSCs without significant changes over treatment)
[88]	FC	10	pN1 (5 T3a/b N1)	RT (8)	Both CD14+ monocytic and CD14- granulocytic MDSCs expressed PD-L1 and PD-L2; their expression was 2-fold greater in CD14- granulocytic MDSCs. Granulocytic MDSCs probably suppressed tumor-reactive CD8+ T cells in metastatic pelvic lymph nodes and exhibited high expression of immune checkpoint molecules in nodal metastases.
[87]	NR	32 PCs 25 HDs	NR	NR	Significant increase of MDSCs (*p* < 0.01), Arg-1, iNOS, and PD-L1 peripheral blood levels in PC patients. Significant differences in distribution of CD14+ monocytic MDSCs and CD15+ polymorphonuclear MDSCs subsets between the 2 groups (60.4% vs. 72.2%, 29.5% vs. 18.8%) (*p* < 0.05). The percentage of MDSCs and monocytic MDSCs correlated to the total survival rate of PC patients (*p* = 0.025; *p* = 0.017).
[96]	FC	15(?)	M1	ENZ	Increased frequency of PD-L1/2+ DCs in CRPC patients progressing on ENZ compared to responders (*p* = 0.006) or naïve groups (*p* = 0.0037). In progressing patients, more PD-L1/2+ DCs were associated with poorer ENZ-response and treatment duration. Initial responders (<50% decrease in PSA) had more circulating PD-L1/2+ DCs.
[97]	FC	NR	NR	NR	CD14+ TILs of PC biopsies expressed higher levels of PD-L1 and PD-1 (vs. lymphocytes isolated from PBMCs of HDs). PD-L1+ myeloid cells seemed suppressors of TILs.
[101]	FC	15 PCs HDs (unclear number)	N1 or M1	Variable (no, hormones, RT, Ticilimumab)	PD-1/PD-L1 blockade (not PD-L2) enhanced Staphylococcus Enterotoxin B-induced IL-2 production in HDs, shifting the antigen-induced cellular reactivity toward a pro-inflammatory Th1/Th17 response (increased IFN-γ, IL-2, TNF-α, IL-6, and IL-17; reduced IL-5, IL-13).
[54]	FC	4	M1	Dur + Ola to mCRPCs (prior ENZ/ABT)	Similar frequencies of PD-L1 expression in T (CD4+, CD8+), B, NK, dendritic, and myeloid-derived suppressor cells (PBMC samples) as described in advanced cancer [156]

Note: in all the studies, the Grade Group/Gleason score of the cases was unavailable/unclear. (*): standard arm: external beam radiotherapy 2.0–2.7 Gy daily fractions; short-course arm: stereotactic body radiotherapy 7.4 Gy every other day or high dose rate brachytherapy 13.5 Gy weekly. Cohorts were balanced as to age, Gleason score, and pre-treatment PSA. ABT, abiraterone; CRPC, castration-resistant prostate cancer; DCs, dendritic cells; Dur, durvalumab; ENZ, enzalutamide; FC, flow cytometry; HDs, healthy donors; IR, intermediate-risk prostate cancer; LR, low-risk prostate cancer; MDSC, myeloid-derived suppressor cells; mCRPC, metastatic castration-resistant prostate cancer; NR, not reported; Ola, Olaparib; PC, prostate cancer; PBMC, peripheral blood mononuclear cells; Ref., reference; RT, radiotherapy; TILs, tumor-infiltrating lymphocytes.

## References

[1] Dai S., Jia R., Zhang X., Fang Q., Huang L. (2014). The PD-1/PD-Ls pathway and autoimmune diseases. Cell. Immunol..

[2] Patsoukis N., Wang Q., Strauss L., Boussiotis V.A. (2020). Revisiting the PD-1 pathway. Sci. Adv..

[3] Santandrea G., Piana S., Valli R., Zanelli M., Gasparini E., De Leo A., Mandato V., Palicelli A. (2021). Immunohistochemical Biomarkers as a Surrogate of Molecular Analysis in Ovarian Carcinomas: A Review of the Literature. Diagnostics.

[4] National Comprehensive Cancer Network (NCCN) Clinical Practice Guidelines in Oncology Prostate Cancer. Version 2.2021—17 February. https://www.nccn.org/professionals/physician_gls/pdf/prostate.pdf.

[5] Foda A.A., Palicelli A., Shebl A., Boldorini R., Elnaghi K., ElHawary A.K. (2019). Role of ERCC1 expression in colorectal adenoma-carcinoma sequence and relation to other mismatch repair proteins expression, clinicopathological features and prognosis in mucinous and non-mucinous colorectal carcinoma. Indian J. Pathol. Microbiol..

[6] De Leo A., Santini D., Ceccarelli C., Santandrea G., Palicelli A., Acquaviva G., Chiarucci F., Rosini F., Ravegnini G., Pession A. (2021). What Is New on Ovarian Carcinoma: Integrated Morphologic and Molecular Analysis Following the New 2020 World Health Organization Classification of Female Genital Tumors. Diagnostics.

[7] Heitzer E., Haque I.S., Roberts C.E.S., Speicher M.R. (2018). Current and future perspectives of liquid biopsies in genomics-driven oncology. Nat. Rev. Genet..

[8] Sharma P., Pachynski R.K., Narayan V., Fléchon A., Gravis G., Galsky M.D., Mahammedi H., Patnaik A., Subudhi S.K., Ciprotti M. (2020). Nivolumab Plus Ipilimumab for Metastatic Castration-Resistant Prostate Cancer: Preliminary Analysis of Patients in the CheckMate 650 Trial. Cancer Cell.

[9] Antonarakis E.S., Piulats J.M., Gross-Goupil M., Goh J., Ojamaa K., Hoimes C.J., Vaishampayan U., Berger R., Sezer A., Alanko T. (2020). Pembrolizumab for Treatment-Refractory Metastatic Castration-Resistant Prostate Cancer: Multicohort, Open-Label Phase II KEYNOTE-199 Study. J. Clin. Oncol..

[10] Zhou Q., Chen X., He H., Peng S., Zhang Y., Zhang J., Cheng L., Liu S., Huang M., Xie R. (2021). WD repeat domain 5 promotes chemoresistance and Programmed Death-Ligand 1 expression in prostate cancer. Theranostics.

[11] Vardaki I., Corn P., Gentile E., Song J.H., Madan N., Hoang A., Parikh N.U., Guerra L.D., Lee Y.-C., Lin S.-C. (2021). Radium-223 Treatment Increases Immune Checkpoint Expression in Extracellular Vesicles from the Metastatic Prostate Cancer Bone Microenvironment. Clin. Cancer Res..

[12] Shim K.H., Kwon J.E., Park S.G., Choo S.H., Kim S.J., Kim S.I. (2021). Cell membrane and nuclear expression of programmed death ligand-1 in prostate needle biopsy tissue in prostate cancer patients undergoing primary radiation therapy. Urol. Oncol. Semin. Orig. Investig..

[13] Sun Y., Jing J., Xu H., Xu L., Hu H., Tang C., Liu S., Wei Q., Duan R., Guo J. (2021). N-cadherin inhibitor creates a microenvironment that protect TILs from immune checkpoints and Treg cells. J. Immunother. Cancer.

[14] Zavridou M., Strati A., Bournakis E., Smilkou S., Tserpeli V., Lianidou E. (2021). Prognostic Significance of Gene Expression and DNA Methylation Markers in Circulating Tumor Cells and Paired Plasma Derived Exosomes in Metastatic Castration Resistant Prostate Cancer. Cancers.

[15] Brady L., Kriner M., Coleman I., Morrissey C., Roudier M., True L.D., Gulati R., Plymate S.R., Zhou Z., Birditt B. (2021). Inter- and intra-tumor heterogeneity of metastatic prostate cancer determined by digital spatial gene expression profiling. Nat. Commun..

[16] Zhang T., Agarwal A., Almquist R.G., Runyambo D., Park S., Bronson E., Boominathan R., Rao C., Anand M., Oyekunle T. (2021). Expression of immune checkpoints on circulating tumor cells in men with metastatic prostate cancer. Biomark. Res..

[17] Petrylak D.P., Loriot Y., Shaffer D.R., Braiteh F., Powderly J., Harshman L.C., Conkling P., Delord J.P., Gordon M., Kim J.W. (2021). Safety and Clinical Activity of Atezolizumab in Patients with Metastatic Castration-Resistant Prostate Cancer: A Phase I Study. Clinical cancer research: An official journal of the American Association for Cancer Research 2021. Clin. Cancer Res..

[18] Imamura R., Kitagawa S., Kubo T., Irie A., Kariu T., Yoneda M., Kamba T., Imamura T. (2020). Prostate cancer C5a receptor expression and augmentation of cancer cell proliferation, invasion, and PD-L1 expression by C5a. Prostate.

[19] Meng J., Zhou Y., Lu X., Bian Z., Chen Y., Zhou J., Zhang L., Hao Z., Zhang M., Liang C. (2020). Immune response drives outcomes in prostate cancer: Implications for immunotherapy. Mol. Oncol..

[20] Wong J.K., MacFarlane A., Devarajan K., Shulman R.M., Alpaugh R.K., Burbure N., Hallman M.A., Geynisman D.M., Horwitz E.M., Campbell K. (2020). Hypofractionated Short Course Radiation Treatment Results in Systemic Immune Activation and Upregulation of the PD-1/PD-L1 Exhaustion Axis: A Prospective Pilot Study in Early Stage Prostate Cancer Patients. Int. J. Radiat. Oncol. Biol. Phys..

[21] Graff J.N., Beer T.M., Alumkal J.J., Slottke R.E., Redmond W.L., Thomas G.V., Thompson R.F., Wood M.A., Koguchi Y., Chen Y. (2020). A phase II single-arm study of pembrolizumab with en-zalutamide in men with metastatic castration-resistant prostate cancer progressing on enzalutamide alone. J. Immunother. Cancer.

[22] Chen Q.-H., Li B., Liu D.-G., Zhang B., Yang X., Tu Y.-L. (2020). LncRNA KCNQ1OT1 sponges miR-15a to promote immune evasion and malignant progression of prostate cancer via up-regulating PD-L. Cancer Cell Int..

[23] Wang Q., Ye Y., Yu H., Lin S.-H., Tu H., Liang D., Chang D.W., Huang M., Wu X. (2020). Immune checkpoint-related serum proteins and genetic variants predict outcomes of localized prostate cancer, a cohort study. Cancer Immunol. Immunother..

[24] Han H.J., Li Y.R., Roach M., Aggarwal R. (2020). Dramatic response to combination pembrolizumab and radiation in meta-static castration resistant prostate cancer. Ther. Adv. Med Oncol..

[25] Vicier C., Ravi P., Kwak L., Ms L.W., Huang Y., Evan C., Loda M., Hamid A.A., Sweeney C.J. (2020). Association between CD8 and PD-L1 expression and outcomes after radical prostatectomy for localized prostate cancer. Prostate.

[26] Ryan S.T., Zhang J., Burner D.N., Liss M., Pittman E., Muldong M., Shabaik A., Woo J., Basler N., Cunha J. (2020). Neoadjuvant rituximab modulates the tumor immune environment in patients with high risk prostate cancer. J. Transl. Med..

[27] Sharma M., Yang Z., Miyamoto H. (2020). Loss of DNA mismatch repair proteins in prostate cancer. Medicine.

[28] Wagle M.-C., Castillo J., Srinivasan S., Holcomb T., Yuen K.C., Kadel E.E., Mariathasan S., Halligan D.L., Carr A.R., Bylesjo M. (2020). Tumor Fusion Burden as a Hallmark of Immune Infiltration in Prostate Cancer. Cancer Immunol. Res..

[29] Obradovic A., Dallos M.C., Zahurak M.L., Partin A.W., Schaeffer E.M., Ross A.E., Allaf M.E., Nirschl T.R., Liu D., Chapman C.G. (2020). T-Cell Infiltration and Adaptive Treg Resistance in Response to Androgen Deprivation with or Without Vaccination in Localized Prostate Cancer. Clin. Cancer Res..

[30] Goswami S., Walle T., Cornish A.E., Basu S., Anandhan S., Fernandez I., Vence L., Blando J., Zhao H., Yadav S.S. (2019). Immune profiling of human tumors identifies CD73 as a combinatorial target in glioblastoma. Nat. Med..

[31] Ihle C., Provera M.D., Straign D.M., Smith E.E., Edgerton S.M., Van Bokhoven A., Lucia M.S., Owens P. (2019). Distinct tumor microenvironments of lytic and blastic bone metastases in prostate cancer patients. J. Immunother. Cancer.

[32] Ross A.E., Hurley P.J., Tran P.T., Rowe S.P., Benzon B., Neal T.O., Chapman C., Harb R., Milman Y., Trock B.J. (2020). A pilot trial of pembrolizumab plus prostatic cryotherapy for men with newly diagnosed oligometa-static hormone-sensitive prostate cancer. Prostate Cancer Prostatic Dis..

[33] Bryce A.H., Dronca R.S., Costello B.A., Infante J.R., Ames T.D., Jimeno J., Karp D.D. (2020). PT-112 in advanced metastatic castrate-resistant prostate cancer (mCRPC), as monotherapy or in combination with PD-L1 inhibitor avelumab: Findings from two phase I studies. J. Clin. Oncol..

[34] Abdul Sater H., Marté J.L., Donahue R.N., Walter-Rodriguez B., Heery C.R., Steinberg S.M., Cordes L.M., Chun G., Karzai F., Bilusic M. (2020). Neoadjuvant PROSTVAC prior to radical prostatectomy enhances T-cell infiltration into the tumor immune microenvi-ronment in men with prostate cancer. J. Immunother. Cancer.

[35] Sharma M., Yang Z., Miyamoto H. (2019). Immunohistochemistry of immune checkpoint markers PD-1 and PD-L1 in prostate cancer. Medicine.

[36] Shaw K.C., Calagua C., Russo J., Einstein D., Balk S., Ye H. (2019). Tumor PD-L1 Expression is Detected in a Significant Subset of High-Risk Localized and Metastatic Prostate Cancer but is Rare in Ductal Subtype. Abstracts from USCAP 2019: Genitourinary Pathology (including renal tumors) (776-992). Modern Pathol..

[37] Matveev V.M., Kirichek A.K., Safronova V.S., Khafizov K.K., Filippova M.F., Lyubchenko L.L. (2019). [Impact of PD-L1 status on the long-term outcomes of radical treatment of patients with prostate cancer]. Urologiia.

[38] Matveev V.B., Kirichek A.A., Safronova V.M., Kokosadze N.V., Khalmurzaev O.A., Kamolov B.S., Liubchenko L.N. (2019). The prognostic value of tumor PD-L1 status in patients with metastatic prostate cancer. Cancer Urol..

[39] Iacovelli R., Ciccarese C., Brunelli M., Bogina G., Munari E., Bimbatti D., Mosillo C., Fantinel E., Bria E., Martignoni G. (2019). PD-L1 Expression in De Novo Metastatic Castration-sensitive Prostate Cancer. J. Immunother..

[40] Kazantseva M., Mehta S., Eiholzer R.A., Gimenez G., Bowie S., Campbell H., Reily-Bell A.L., Roth I., Ray S., Drummond C.J. (2019). The Δ133p53β isoform promotes an immunosuppressive environment leading to aggressive prostate cancer. Cell Death Dis..

[41] Lindh C., Kis L., Delahunt B., Samaratunga H., Yaxley J., Wiklund N.P., Clements M., Egevad L., Wiklund P. (2019). PD -L1 expression and deficient mismatch repair in ductal adenocarcinoma of the prostate. APMIS.

[42] Richardsen E., Andersen S., Al-Saad S., Rakaee M., Nordby Y., Pedersen M.I., Ness N., Ingebriktsen L.M., Fassina A., Taskén K.A. (2019). Low Expression of miR-424-3p is Highly Correlated with Clinical Failure in Prostate Cancer. Sci. Rep..

[43] Xian P., Ge N., Wu V.J., Patel A., Tang W.W., Wu X., Zhang K., Li L., You Z. (2019). PD-L1 instead of PD-1 status is associated with the clinical features in human primary prostate tumors. Am. J. Clin. Exp. Urol..

[44] Li H., Wang Z., Zhang Y., Sun G., Ding B., Yan L., Liu H., Guan W., Hu Z., Wang S. (2019). The Immune Checkpoint Regulator PDL1 is an Independent Prognostic Biomarker for Biochemical Recurrence in Prostate Cancer Patients Following Adjuvant Hormonal Therapy. J. Cancer.

[45] Pal S.K., Moreira D., Won H., White S.W., Duttagupta P., Lucia M., Jones J., Hsu J., Kortylewski M. (2019). Reduced T-cell Numbers and Elevated Levels of Immunomodulatory Cytokines in Metastatic Prostate Cancer Patients De Novo Resistant to Abiraterone and/or Enzalutamide Therapy. Int. J. Mol. Sci..

[46] Abida W., Cheng M.L., Armenia J., Middha S., Autio K.A., Vargas H.A., Rathkopf D., Morris M.J., Danila D.C., Slovin S.F. (2019). Analysis of the Prevalence of Microsatellite Instability in Prostate Cancer and Response to Immune Checkpoint Blockade. JAMA Oncol..

[47] Zhao S.G., Lehrer J., Chang S.L., Das R., Erho N., Liu Y., Sjöström M., Den R.B., Freedland S.J., Klein E.A. (2019). The Immune Landscape of Prostate Cancer and Nomination of PD-L2 as a Potential Therapeutic Target. J. Natl. Cancer Inst..

[48] Scimeca M., Bonfiglio R., Urbano N., Cerroni C., Anemona L., Montanaro M., Fazi S., Schillaci O., Mauriello A., Bonanno E. (2019). Programmed death ligand 1 expression in prostate cancer cells is associated with deep changes of the tumor inflammatory infiltrate composition. Urol. Oncol. Semin. Orig. Investig..

[49] Jung K.H., LoRusso P., Burris H., Gordon M., Bang Y.-J., Hellmann M.D., Cervantes A., de Olza M.O., Marabelle A., Hodi F.S. (2019). Phase I Study of the Indoleamine 2,3-Dioxygenase 1 (IDO1) Inhibitor Navoximod (GDC-0919) Administered with PD-L1 Inhibitor (Atezolizumab) in Advanced Solid Tumors. Clin. Cancer Res..

[50] Mo R., Han Z., Liang Y., Ye J., Wu S., Lin S.X., Zhang Y., Song S., Jiang F., Zhong W. (2018). Expression of PD-L1 in tumor-associated nerves correlates with reduced CD8+tumor-associated lymphocytes and poor prognosis in prostate cancer. Int. J. Cancer.

[51] Papanicolau-Sengos M.A., Yang Y., Pabla S., Lenzo F.L., Kato S., Kurzrock R., DePietro P., Nesline M., Bs J.C., Glenn S. (2018). Identification of targets for prostate cancer immunotherapy. Prostate.

[52] Von Hardenberg J., Hartmann S., Nitschke K., Worst T., Ting S., Reis H., Nuhn P., Weis C.-A., Erben P. (2018). Programmed Death Ligand 1 (PD-L1) Status and Tumor-Infiltrating Lymphocytes in Hot Spots of Primary and Liver Metastases in Prostate Cancer With Neuroendocrine Differentiation. Clin. Genitourin. Cancer.

[53] Jin X., Ding D., Yan Y., Li H., Wang B., Ma L., Ye Z., Ma T., Wu Q., Rodrigues D.N. (2019). Phosphorylated RB Promotes Cancer Immunity by In-hibiting NF-κB Activation and PD-L1 Expression. Mol. Cell.

[54] Karzai F., VanderWeele D., Madan R.A., Owens H., Cordes L.M., Hankin A., Couvillon A., Nichols E., Bilusic M., Beshiri M. (2018). Activity of durvalumab plus olaparib in metastatic castration-resistant prostate cancer in men with and without DNA damage repair mutations. J. Immunother. Cancer.

[55] Richter I., Jirasek T., Havlickova I., Curcikova R., Samal V., Dvorak J., Bartos J. (2019). The expression of PD-L1 in patients with castrate prostate cancer treated with enzalutamide. JBUON Off. J. Balk. Union Oncol..

[56] Xiong W., Deng H., Huang C., Zen C., Jian C., Ye K., Zhong Z., Zhao X., Zhu L. (2018). MLL3 enhances the transcription of PD-L1 and regulates anti-tumor immunity. Biochim. et Biophys. Acta (BBA)—Mol. Basis Dis..

[57] Hahn E., Liu S., Vesprini D., Xu B., Downes M.R. (2018). Immune infiltrates and PD-L1 expression in treatment-naïve acinar prostatic adenocarcinoma: An exploratory analysis. J. Clin. Pathol..

[58] Redman J.M., Steinberg S.M., Gulley J.L. (2018). Quick efficacy seeking trial (QuEST1): A novel combination immunotherapy study designed for rapid clinical signal assessment metastatic castration-resistant prostate cancer. J. Immunother. Cancer.

[59] Rodrigues D.N., Rescigno P., Liu D., Yuan W., Carreira S., Lambros M.B., Seed G., Mateo J., Riisnaes R., Mullane S. (2018). Immunogenomic analyses associate immunological alterations with mismatch repair defects in prostate cancer. J. Clin. Investig..

[60] Salvi S., Casadio V., Martignano F., Gurioli G., Tumedei M.M., Calistri D., Gunelli R., Costantini M. (2018). Carcinosarcoma of the prostate: Case report with molecular and histological characterization. Int. J. Biol. Markers.

[61] Wang C., Hahn E., Slodkowska E., Eskander A., Enepekides D., Higgins K., Vesprini D., Liu S.K., Downes M.R., Xu B. (2018). Reproducibility of PD-L1 immunohistochemistry interpretation across various types of genitourinary and head/neck carci-nomas, antibody clones, and tissue types. Hum. Pathol..

[62] Hansen A.R., Massard C., Ott P.A., Haas N.B., Lopez J.S., Ejadi S., Wallmark J.M., Keam B., Delord J.P., Aggarwal R. (2018). Pembrolizumab for advanced prostate adenocarcinoma: Findings of the KEYNOTE-028 study. Ann. Oncol. Off. J. Eur. Soc. Med Oncol..

[63] McNeel D.G., Eickhoff J.C., Wargowski E., Zahm C., Staab M.J., Straus J., Liu G. (2018). Concurrent, but not sequential, PD-1 blockade with a DNA vaccine elicits anti-tumor responses in patients with metastatic, castration-resistant prostate cancer. Oncotarget.

[64] Ishiba T., Hoffmann A.-C., Usher J., Elshimali Y., Sturdevant T., Dang M., Jaimes Y., Tyagi R., Gonzales R., Grino M. (2018). Frequencies and expression levels of programmed death ligand 1 (PD-L1) in circulating tumor RNA (ctRNA) in various cancer types. Biochem. Biophys. Res. Commun..

[65] Xu L.J., Ma Q., Zhu J., Li J., Xue B.X., Gao J., Sun C.Y., Zang Y.C., Zhou Y.B., Yang D.R. (2018). Combined in-hibition of JAK1,2/Stat3-PD-L1 signaling pathway suppresses the immune escape of castration-resistant prostate cancer to NK cells in hypoxia. Mol. Med. Rep..

[66] Haffner M.C., Guner G., Taheri D., Netto G.J., Palsgrove D.N., Zheng Q., Guedes L.B., Kim K., Tsai H., Esopi D.M. (2018). Comprehensive Evaluation of Programmed Death-Ligand 1 Expression in Pri-mary and Metastatic Prostate Cancer. Am. J. Pathol..

[67] Nagaputra J., Thike A.A., Koh V. (2018). Loss of Androgen Receptor Accompained by Paucity of PD-L1 in Prostate Cancer is As-sociated with Clinical Relapse. USCAP 2018 Abstracts: Genitourinary Pathology (894–1126). Meet. Abstr. Modern Pathol..

[68] Tu Y.N., Tong W.L., Yavorski J.M., Blanck G. (2018). Immunogenomics: A Negative Prostate Cancer Outcome Associated with TcR-γ/δ Recombinations. Cancer Microenviron. Off. J. Int. Cancer Microenviron. Soc..

[69] Tao Z., Xu S., Ruan H., Wang T., Song W., Qian L., Chen K. (2018). MiR-195/-16 Family Enhances Radiotherapy via T Cell Ac-tivation in the Tumor Microenvironment by Blocking the PD-L1 Immune Checkpoint. Cell. Physiol. Biochem..

[70] Budczies J., Denkert C., Győrffy B., Schirmacher P., Stenzinger A. (2017). Chromosome 9p copy number gains involving PD-L1 are associated with a specific proliferation and immune-modulating gene expression program active across major cancer types. BMC Med Genom..

[71] Fankhauser C.D., Schüffler P.J., Gillessen S., Omlin A., Rupp N.J., Rueschoff J.H., Hermanns T., Poyet C., Sulser T., Moch H. (2017). Comprehensive immunohistochemical analysis of PD-L1 shows scarce expression in castration-resistant prostate cancer. Oncotarget.

[72] Truillet C., Oh H.L.J., Yeo S.P., Lee C.-Y., Huynh L.T., Wei J., Parker M.F.L., Blakely C., Sevillano N., Wang Y.-H. (2017). Imaging PD-L1 Expression with ImmunoPET. Bioconjugate Chem..

[73] Zhang J., Bu X., Wang H., Zhu Y., Geng Y., Nihira N.T., Tan Y., Ci Y., Wu F., Dai X. (2017). Cyclin D–CDK4 kinase destabilizes PD-L1 via cullin 3–SPOP to control cancer immune surveillance. Nature.

[74] Chen Y., Zhang Y., Lv J.-W., Li Y.-Q., Wang Y.-Q., He Q.-M., Yang X.-J., Sun Y., Mao Y.-P., Yun J.-P. (2017). Genomic Analysis of Tumor Microenvironment Immune Types across 14 Solid Cancer Types: Immunotherapeutic Implications. Theranostics.

[75] Calagua C., Russo J., Sun Y., Schaefer R., Lis R., Zhang Z., Mahoney K., Bubley G.J., Loda M., Taplin M.-E. (2017). Expression of PD-L1 in Hormone-naïve and Treated Prostate Cancer Patients Receiving Neoadjuvant Abiraterone Acetate plus Prednisone and Leuprolide. Clin. Cancer Res..

[76] Schott D.S., Pizon M., Pachmann U., Pachmann K. (2017). Sensitive detection of PD-L1 expression on circulating epithelial tumor cells (CETCs) could be a potential biomarker to select patients for treatment with PD-1/PD-L1 inhibitors in early and metastatic solid tumors. Oncotarget.

[77] Petitprez F., Fossati N., Vano Y., Freschi M., Becht E., Lucianò R., Calderaro J., Guédet T., Lacroix L., Rancoita P. (2017). PD-L1 Expression and CD8+ T-cell Infiltrate are Associated with Clinical Progression in Patients with Node-positive Prostate Cancer. Eur. Urol. Focus.

[78] Li G., Ross J., Yang X. (2019). Mismatch Repair (MMR) Deficiency and PD-L1 Expression in the Prostatic Ductal Adenocarcinoma. Abstracts from USCAP 2019: Genitourinary Pathology (including renal tumors) (776–992). Meet. Abstr. Mod. Pathol..

[79] Ness N., Andersen S., Khanehkenari M.R., Nordbakken C.V., Valkov A., Paulsen E.-E., Nordby Y., Bremnes R.M., Donnem T., Busund L.-T. (2017). The prognostic role of immune checkpoint markers programmed cell death protein 1 (PD-1) and programmed death ligand 1 (PD-L1) in a large, multicenter prostate cancer cohort. Oncotarget.

[80] Baas W., Gershburg S., Dynda D., Delfino K., Robinson K., Nie D., Yearley J.H., Alanee S. (2017). Immune Characterization of the Programmed Death Receptor Pathway in High Risk Prostate Cancer. Clin. Genitourin. Cancer.

[81] Gao J., Ward J., A Pettaway C., Shi L.Z., Subudhi S.K., Vence L.M., Zhao H., Chen J., Chen H., Efstathiou E. (2017). VISTA is an inhibitory immune checkpoint that is increased after ipilimumab therapy in patients with prostate cancer. Nat. Med..

[82] Lu X., Horner J.W., Paul E., Shang X., Troncoso P., Deng P., Jiang S., Chang Q., Spring D.J., Sharma P. (2017). Effective combinatorial immunotherapy for castration-resistant prostate cancer. Nature.

[83] Tretiakova M., Fulton R., Kocherginsky M. (2017). Comparison of 4 PD-L1 Antibodies in 560 Kidney, Bladder and Prostate Cancers. Abstracts from USCAP 2019: Genitourinary Pathology (including Renal tumors). Meet. Abstr. Mod. Pathol..

[84] Najjar S.N., Kallakury B.V.S., Sheehan C.E. (2017). Infrequent PD-L1 Protetin Expression and Gene Amplification in Prostatic Adenocarcinomas (PACs). Abstracts from USCAP 2019: Genitourinary Pathology (including Renal tumors). Meet. Abstr. Mod. Pathol..

[85] Hashimoto Y., Imai A., Hatakeyama S., Yoneyama T., Koie T., Ohyama C. (2016). 291P PD-L1 over expression may predict disease aggressiveness in prostate cancer. Ann. Oncol..

[86] Gevensleben H., Holmes E.E., Goltz D., Dietrich J., Sailer V., Ellinger J., Dietrich D., Kristiansen G. (2016). PD-L1promoter methylation is a prognostic biomarker for biochemical recurrence-free survival in prostate cancer patients following radical prostatectomy. Oncotarget.

[87] Zhou Q.-Z., Liu C.-D., Yang J.-K., Guo W.-B., Zhou J.-H., Bian J. (2016). [Changed percentage of myeloid-derived suppressor cells in the peripheral blood of prostate cancer patients and its clinical implication]. Zhonghua Nan ke Xue = Natl. J. Androl..

[88] Sharma V., Dong H., Kwon E., Karnes R.J. (2016). Positive Pelvic Lymph Nodes in Prostate Cancer Harbor Immune Suppressor Cells to Impair Tumor-reactive T Cells. Eur. Urol. Focus.

[89] Goltz D., Holmes E.E., Gevensleben H., Sailer V., Dietrich J., Jung M., Röhler M., Meller S., Ellinger J., Kristiansen G. (2016). CXCL12 promoter methylation and PD-L1 expression as prognostic biomarkers in prostate cancer patients. Oncotarget.

[90] Graff J.N., Alumkal J.J., Drake C.G., Thomas G., Redmond W., Farhad M., Cetnar J.P., Ey F.S., Bergan R.C., Slottke R. (2016). Early evidence of anti-PD-1 activity in enzalutamide-resistant prostate cancer. Oncotarget.

[91] Satelli A., Batth I.S., Brownlee Z., Rojas C., Meng Q.H., Kopetz S., Li S. (2016). Potential role of nuclear PD-L1 expression in cell-surface vimentin positive circulating tumor cells as a prognostic marker in cancer patients. Sci. Rep..

[92] Massari F., Ciccarese C., Caliò A., Munari E., Cima L., Porcaro A., Novella G., Artibani W., Sava T., Eccher A. (2015). Magnitude of PD-1, PD-L1 and T Lymphocyte Expression on Tissue from Castration-Resistant Prostate Adenocarcinoma: An Exploratory Analysis. Target. Oncol..

[93] Gevensleben H., Dietrich D., Golletz C., Steiner S., Jung M., Thiesler T., Majores M., Stein J., Uhl B., Müller S. (2016). The Immune Checkpoint Regulator PD-L1 Is Highly Expressed in Aggressive Primary Prostate Cancer. Clin. Cancer Res..

[94] Martin A.M., Nirschl T.R., Nirschl C.J., Francica B.J., Kochel C.M., Van Bokhoven A., Meeker A.K., Lucia M.S., Anders R.A., DeMarzo A.M. (2015). Paucity of PD-L1 expression in prostate cancer: Innate and adaptive immune resistance. Prostate Cancer Prostatic Dis..

[95] Shalapour S., Font-Burgada J., Di Caro G., Zhong Z., Sanchez-Lopez E., Dhar D., Willimsky G., Ammirante M., Strasner A., Hansel D.E. (2015). Immunosuppressive plasma cells impede T-cell-dependent immunogenic chemotherapy. Nature.

[96] Bishop J.L., Sio A., Angeles A., Roberts M.E., Azad A.A., Chi K.N., Zoubeidi A. (2015). PD-L1 is highly expressed in Enzalu-tamide resistant prostate cancer. Oncotarget.

[97] Spary L., Salimu J., Webber J.P., Clayton A., Mason M.D., Tabi Z. (2014). Tumor stroma-derived factors skew monocyte to dendritic cell differentiation toward a suppressive CD14+PD-L1+phenotype in prostate cancer. OncoImmunology.

[98] Taube J.M. (2014). Unleashing the immune system: PD-1 and PD-Ls in the pre-treatment tumor microenvironment and correlation with response to PD-1/PD-L1 blockade. Oncoimmunology.

[99] Taube J.M., Klein A., Brahmer J.R., Xu H., Pan X., Kim J.H., Chen L., Pardoll D.M., Topalian S.L., Anders R.A. (2014). As-sociation of PD-1, PD-1 ligands, and other features of the tumor immune microenvironment with response to anti-PD-1 therapy. Clin. Cancer Res..

[100] Topalian S.L., Hodi F.S., Brahmer J.R., Gettinger S.N., Smith D.C., McDermott D.F., Powderly J.D., Carvajal R.D., Sosman J.A., Atkins M.B. (2012). Safety, activity, and immune correlates of anti-PD-1 antibody in cancer. New Engl. J. Med..

[101] Dulos J., Carven G.J., van Boxtel S.J., Evers S., Driessen-Engels L.J.A., Hobo W., Gorecka M.A., de Haan A.F.J., Mulders P., Punt C.J.A. (2012). PD-1 Blockade Augments Th1 and Th17 and Suppresses Th2 Responses in Peripheral Blood from Patients With Prostate and Advanced Melanoma Cancer. J. Immunother..

[102] Zhou J.E., Yu J., Wang Y., Wang H., Wang J., Wang Y., Yu L., Yan Z. (2021). ShRNA-mediated silencing of PD-1 augments the efficacy of chimeric antigen receptor T cells on subcutaneous prostate and leukemia xenograft. Biomed. Pharmacother..

[103] Wu Y., Xie J., Jin X., Lenchine R.V., Wang X., Fang D.M., Nassar Z.D., Butler L.M., Li J., Proud C.G. (2020). eEF2K enhances expression of PD-L1 by promoting the translation of its mRNA. Biochem. J..

[104] Zhang W., Shi X., Chen R., Zhu Y., Peng S., Chang Y., Nian X., Xiao G., Fang Z., Li Y. (2020). Novel Long Non-coding RNA lncAMPC Promotes Metastasis and Immunosuppression in Prostate Cancer by Stimu-lating LIF/LIFR Expression. J. Am. Soc. Gene Ther..

[105] Rennier K.R., Shin W.J., Krug E., Virdi G.S., Pachynski R.K. (2020). Chemerin Reactivates PTEN and Suppresses PD-L1 in Tumor Cells via Modulation of a Novel CMKLR1-mediated Signaling Cascade. Clin. Cancer Res..

[106] Philippou Y., Sjoberg H.T., Murphy E., Alyacoubi S., Jones K.I., Gordon-Weeks A.N., Phyu S., Parkes E.E., Gillies McKenna W., Lamb A.D. (2020). Impacts of combining anti-PD-L1 immunotherapy and radiotherapy on the tumour immune mi-croenvironment in a murine prostate cancer model. Br. J. Cancer.

[107] Wang B., Sun L., Yuan Z., Tao Z. (2020). Wee1 kinase inhibitor AZD1775 potentiates CD8+ T cell-dependent antitumour activity via dendritic cell activation following a single high dose of irradiation. Med Oncol..

[108] Papaevangelou E., Smolarek D., A Smith R., Dasgupta P., Galustian C. (2020). Targeting Prostate Cancer Using Intratumoral Cytotopically Modified Interleukin-15 Immunotherapy in a Syngeneic Murine Model. ImmunoTargets Ther..

[109] Wei J., Wang Y.-H., Lee C.Y., Truillet C., Oh D.Y., Xu Y., Ruggero D., Flavell R.R., VanBrocklin H.F., Seo Y. (2020). An Analysis of Isoclonal Antibody Formats Suggests a Role for Measuring PD-L1 with Low Molecular Weight PET Radiotracers. Mol. Imaging Biol..

[110] Ding H., Wang Z., Pascal Laura E., Chen W., Wang Z., Wang Z. (2020). ELL2 DEFICIENCY UPREGULATES PD-L1 EXPRESSION VIA JAK2 SIGNALING IN PROSTATE CANCER CELLS. Meeting Abstract: MP51. J. Urol..

[111] Sun Y., Wei Q., Huang J., Yang L. (2020). MP16-12 METHYLATION CAN REGULATE THE EXPRESSION OF PD-L1 IN SMALL CELL PROSTATE CANCER. J. Urol..

[112] Liu J., He D., Cheng L., Huang C., Zhang Y., Rao X., Kong Y., Li C., Zhang Z., Liu J. (2020). p300/CBP inhibition enhances the efficacy of programmed death-ligand 1 blockade treatment in prostate cancer. Oncogene.

[113] Yamazaki T., Buqué A., Ames T.D., Galluzzi L. (2020). PT-112 induces immunogenic cell death and synergizes with immune checkpoint blockers in mouse tumor models. OncoImmunology.

[114] Zhang X., Chen H., Li G., Zhou X., Shi Y., Zou F., Chen Y., Gao J., Yang S., Wu S. (2020). Increased Tim-3 expression on TILs during treatment with the Anchored GM-CSF vaccine and anti-PD-1 antibodies is inversely correlated with response in prostate cancer. J. Cancer.

[115] Wang B., Zhou Y., Zhang J., Jin X., Wu H., Huang H. (2020). Fructose-1,6-bisphosphatase loss modulates STAT3-dependent ex-pression of PD-L1 and cancer immunity. Theranostics.

[116] Orme J.J., Jazieh K.A., Xie T., Harrington S., Liu X., Ball M., Madden B., Charlesworth M.C., Azam T.U., Lucien F. (2020). ADAM10 and ADAM17 cleave PD-L1 to mediate PD-(L)1 inhibitor resistance. OncoImmunology.

[117] Gan S., Ye J., Li J., Hu C., Wang J., Xu D., Pan X., Chu C., Chu J., Zhang J. (2019). LRP11 activates β-catenin to induce PD-L1 expression in prostate cancer. J. Drug Target..

[118] Choi B., Jung H., Yu B., Choi H., Lee J., Kim D. (2019). Sequential MR Image-Guided Local Immune Checkpoint Blockade Cancer Immunotherapy Using Ferumoxytol Capped Ultralarge Pore Mesoporous Silica Carriers after Standard Chemotherapy. Small.

[119] Mao W., Ghasemzadeh A., Freeman Z., Obradovic A., Chaimowitz M.G., Nirschl T.R., McKiernan E., Yegnasubramanian S., Drake C.G. (2019). Immunogenicity of prostate cancer is augmented by BET bromodomain inhibition. J. Immunother. Cancer.

[120] Zhou Q., Xiong W., Zhou X., Gao R.S., Lin Q.F., Liu H.Y., Li J.N., Tian X.F. (2019). CTHRC1 and PD-1/PD-L1 expression predicts tumor recurrence in prostate cancer. Mol. Med. Rep..

[121] Xu Q., Long Q., Zhu D., Fu D., Zhang B., Han L., Qian M., Guo J., Xu J., Cao L. (2019). Targeting amphiregulin (AREG) derived from senescent stromal cells diminishes cancer resistance and averts programmed cell death 1 ligand (PD-L1)-mediated immunosuppression. Aging Cell.

[122] Dudzinski S.O., Cameron B.D., Wang J., Rathmell J.C., Giorgio T.D., Kirschner A.N. (2019). Combination immunotherapy and radiotherapy causes an abscopal treatment response in a mouse model of castration resistant prostate cancer. J. Immunother. Cancer.

[123] Li X., Wang Z., Huang J., Luo H., Zhu S., Yi H., Zheng L., Hu B., Yu L., Li L. (2018). Specific zinc finger-induced methylation of PD-L1 promoter inhibits its expression. FEBS Open Bio.

[124] Liu K., Zhou Z., Gao H., Yang F., Qian Y., Jin H., Guo Y., Liu Y., Li H., Zhang C. (2019). JQ1, a BET-bromodomain inhibitor, inhibits human cancer growth and suppresses PD-L1 expression. Cell Biol. Int..

[125] Xu N., Huang L., Li X., Watanabe M., Li C., Xu A., Liu C., Li Q., Araki M., Wada K. (2019). The Novel Combination of Nitroxoline and PD-1 Blockade, Exerts a Potent Antitumor Effect in a Mouse Model of Prostate Cancer. Int. J. Biol. Sci..

[126] Yoneda T., Kunimura N., Kitagawa K., Fukui Y., Saito H., Narikiyo K., Ishiko M., Otsuki N., Nibu K.I., Fujisawa M. (2019). Overexpression of SOCS3 mediated by adenovirus vector in mouse and human castra-tion-resistant prostate cancer cells increases the sensitivity to NK cells in vitro and in vivo. Cancer Gene Ther..

[127] Fenerty K.E., Padget M., Wolfson B., Gameiro S.R., Su Z., Lee J.H., Rabizadeh S., Soon-Shiong P., Hodge J.W. (2018). Im-munotherapy utilizing the combination of natural killer- and antibody dependent cellular cytotoxicity (ADCC)-mediating agents with poly (ADP-ribose) polymerase (PARP) inhibition. J. Immunother. Cancer.

[128] Krueger T.E., Thorek D.L.J., Meeker A.K., Isaacs J.T., Brennen W.N. (2018). Tumor-infiltrating mesenchymal stem cells: Drivers of the immunosuppressive tumor microenvironment in prostate cancer?. Prostate.

[129] Medina Enríquez M.M., Félix A.J., Ciudad C.J., Noé V. (2018). Cancer immunotherapy using PolyPurine Reverse Hoogsteen hairpins targeting the PD-1/PD-L1 pathway in human tumor cells. PLoS ONE.

[130] Moreira D., Adamus T., Zhao X., Su Y.-L., Zhang Z., White S.V., Swiderski P., Lu X., Depinho R.A., Pal S.K. (2018). STAT3 Inhibition Combined with CpG Immunostimulation Activates Antitumor Immunity to Eradicate Genetically Distinct Castration-Resistant Prostate Cancers. Clin. Cancer Res..

[131] Zhang L., Xu L.J., Zhu J., Li J., Xue B.X., Gao J., Sun C.Y., Zang Y.C., Zhou Y.B., Yang D.R. (2018). ATM-JAK-PD-L1 signaling pathway inhibition decreases EMT and metastasis of androgen-independent prostate cancer. Mol. Med. Rep..

[132] Yin C., Wang Y., Ji J., Cai B., Chen H., Yang Z., Wang K., Luo C., Zhang W.-W., Yuan C. (2018). Molecular Profiling of Pooled Circulating Tumor Cells from Prostate Cancer Patients Using a Dual-Antibody-Functionalized Microfluidic Device. Anal. Chem..

[133] Ahern E., Harjunpää H., O’Donnell J.S., Allen S., Dougall W.C., Teng M.W.L., Smyth M.J. (2018). RANKL blockade improves efficacy of PD1-PD-L1 blockade or dual PD1-PD-L1 and CTLA4 blockade in mouse models of cancer. Oncoimmunology.

[134] Xu L., Shen M., Chen X., Yang D.-R., Tsai Y., Keng P.C., Lee S.O., Chen Y. (2018). In vitro -induced M2 type macrophages induces the resistance of prostate cancer cells to cytotoxic action of NK cells. Exp. Cell Res..

[135] Xu L., Chen X., Shen M., Yang D.R., Fang L., Weng G., Tsai Y., Keng P.C., Chen Y., Lee S.O. (2018). Inhibition of IL-6-JAK/Stat3 signaling in castration-resistant prostate cancer cells enhances the NK cell-mediated cytotoxicity via alteration of PD-L1/NKG2D ligand levels. Mol. Oncol..

[136] Xu L., Shen M., Chen X., Zhu R., Yang D.R., Tsai Y., Keng P.C., Chen Y., Lee S.O. (2018). Adipocytes affect castration-resistant prostate cancer cells to develop the resistance to cytotoxic action of NK cells with alterations of PD-L1/NKG2D ligand levels in tumor cells. Prostate.

[137] Zhang Y., Zhu S., Qian P., Wang X., Xu Z., Sun W., Xu Y. (2019). RelB upregulates PD-L1 in advanced prostate cancer: An insight into tumor immunoescape. Meet. Abstr. Cancer Res..

[138] Shimizu N., Velasco M.A.D., Kura Y. (2018). PD-L1 immune checkpoint blockade in genetically engineered mouse models of prostate cancer. Abstracts of the 76th Annual Meeting of the Japanese Cancer Association; 2017 Sept Kura., Y., 28-30; Yokohama, Japan. Meeting Abstract. P-Cancer Sci..

[139] Maher C.M., Thomas J.D., Haas D., Longen C.G., Oyer H.M., Tong J., Kim F.J. (2017). Small-Molecule Sigma1 Modulator Induces Autophagic Degradation of PD-L. Mol. Cancer Res..

[140] Shi X., Zhang X., Li J., Zhao H., Mo L., Shi X., Hu Z., Gao J., Tan W. (2017). PD-1/PD-L1 blockade enhances the efficacy of SA-GM-CSF surface-modified tumor vaccine in prostate cancer. Cancer Lett..

[141] Cappuccini F., Pollock E., Stribbling S., Hill A.V., Redchenko I. (2017). 5T4 oncofoetal glycoprotein: An old target for a novel prostate cancer immunotherapy. Oncotarget.

[142] Velasco M.A.D., Kura Y., Ando N., Sato N., Sakai K., Davies B.R., Sugimoto K., Nozawa M., Yoshimura K., Yoshikawa K. (2017). PD-L1 blockade in preclinical models of PTEN-deficient prostate cancer. Meeting Abstract. Meeting Abstract. Cancer Res..

[143] Liu Z., Zhao Y., Fang J., Cui R., Xiao Y., Xu Q. (2017). SHP2 negatively regulates HLA-ABC and PD-L1 expression via STAT1 phosphorylation in prostate cancer cells. Oncotarget.

[144] Tanoue K., Shaw A.R., Watanabe N., Porter C., Rana B., Gottschalk S., Brenner M., Suzuki M. (2017). Armed Oncolytic Adenovirus–Expressing PD-L1 Mini-Body Enhances Antitumor Effects of Chimeric Antigen Receptor T Cells in Solid Tumors. Cancer Res..

[145] Wang X., Yang L., Huang F., Zhang Q., Liu S., Ma L., You Z. (2017). Inflammatory cytokines IL-17 and TNF-α up-regulate PD-L1 expression in human prostate and colon cancer cells. Immunol. Lett..

[146] Serganova I., Moroz E., Cohen I., Moroz M., Mane M., Zurita J., Shenker L., Ponomarev V., Blasberg R. (2017). Enhancement of PSMA-Directed CAR Adoptive Immunotherapy by PD-1/PD-L1 Blockade. Mol. Ther. Oncolytics.

[147] Rekoske B.T., Olson B.M., McNeel D.G. (2016). Antitumor vaccination of prostate cancer patients elicits PD-1/PD-L1 regulated antigen-specific immune responses. Oncoimmunology.

[148] Rekoske B.T., Smith H.A., Olson B.M., Maricque B.B., McNeel D.G. (2015). PD-1 or PD-L1 Blockade Restores Antitumor Efficacy Following SSX2 Epitope–Modified DNA Vaccine Immunization. Cancer Immunol. Res..

[149] Black M., Barsoum I.B., Truesdell P., Cotechini T., Macdonald-Goodfellow S.K., Petroff M., Siemens D.R., Koti M., Craig A.W., Graham C.H. (2016). Activation of the PD-1/PD-L1 immune checkpoint confers tumor cell chemoresistance associated with increased metastasis. Oncotarget.

[150] Yang S., Zhang Q., Liu S., Wang A.R., You Z. (2016). PD-1, PD-L1 and PD-L2 expression in mouse prostate cancer. Am. J. Clin. Exp. Urol..

[151] Carbotti G., Barisione G., Airoldi I., Mezzanzanica D., Bagnoli M., Ferrero S., Petretto A., Fabbi M., Ferrini S. (2015). IL-27 induces the expression of IDO and PD-L1 in human cancer cells. Oncotarget.

[152] Bernstein M.B., Garnett C.T., Zhang H., Velcich A., Wattenberg M., Gameiro S.R., Kalnicki S., Hodge J.W., Guha C. (2014). Radiation-Induced Modulation of Costimulatory and Coinhibitory T-Cell Signaling Molecules on Human Prostate Carcinoma Cells Promotes Productive Antitumor Immune Interactions. Cancer Biotherapy Radiopharm..

[153] Yu P., Steel J., Zhang M., Morris J.C., Waitz R., Fasso M., Allison J., Waldmann T.A. (2012). Simultaneous inhibition of two regulatory T-cell subsets enhanced Interleukin-15 efficacy in a prostate tumor model. Proc. Natl. Acad. Sci. USA.

[154] Lin H., Liu Q., Zeng X., Yu W., Xu G. (2021). Pembrolizumab with or without enzalutamide in selected populations of men with previously untreated metastatic castration-resistant prostate cancer harbouring programmed cell death ligand-1 staining: A retrospective study. BMC Cancer.

[155] Morel K.L., Sheahan A.V., Burkhart D.L., Baca S.C., Boufaied N., Liu Y., Qiu X., Cañadas I., Roehle K., Heckler M. (2021). EZH2 inhibition activates a dsRNA-STING-interferon stress axis that potentiates response to PD-1 checkpoint blockade in prostate cancer. Nat. Cancer.

[156] Donahue R.N., Lepone L.M., Grenga I., Jochems C., Fantini M., Madan R.A., Heery C.R., Gulley J.L., Schlom J. (2017). Analyses of the peripheral immunome following multiple administrations of avelumab, a human IgG1 anti-PD-L1 monoclonal antibody. J. Immunother. Cancer.

[157] Won H., Moreira D., Gao C., Duttagupta P., Zhao X., Manuel E., Diamond D., Yuan Y., Liu Z., Jones J. (2017). TLR9 expression and secretion of LIF by prostate cancer cells stimulates accumulation and activity of polymorphonuclear MDSCs. J. Leukoc. Biol..

[158] Harris W.P., Mostaghel E.A., Nelson P.S., Montgomery B. (2009). Androgen deprivation therapy: Progress in understanding mechanisms of resistance and optimizing androgen depletion. Nat. Clin. Pract. Urol..

[159] Tran C., Ouk S., Clegg N.J., Chen Y., Watson P.A., Arora V., Wongvipat J., Smith-Jones P.M., Yoo D., Kwon A. (2009). Development of a Second-Generation Antiandrogen for Treatment of Advanced Prostate Cancer. Science.

[160] Sarnaik A.A., Yu B., Yu D., Morelli D., Hall M., Bogle D., Yan L., Targan S., Solomon J., Nichol G. (2010). Extended Dose Ipilimumab with a Peptide Vaccine: Immune Correlates Associated with Clinical Benefit in Patients with Resected High-Risk Stage IIIc/IV Melanoma. Clin. Cancer Res..

[161] Pagès F., Galon J., Dieu-Nosjean M.-C., Tartour E., Sautes-Fridman C., Fridman W.H. (2009). Immune infiltration in human tumors: A prognostic factor that should not be ignored. Oncogene.

[162] Gedye C., Hussain A., Paterson J., Smrke A., Saini H., Sirskyj D., Pereira K., Lobo N., Stewart J., Go C. (2014). Cell Surface Profiling Using High-Throughput Flow Cytometry: A Platform for Biomarker Discovery and Analysis of Cellular Heterogeneity. PLoS ONE.

[163] Brouwer A., De Laere B., Peeters D., Peeters M., Salgado R., Dirix L., Van Laere S. (2016). Evaluation and consequences of heterogeneity in the circulating tumor cell compartment. Oncotarget.

[164] Zainfeld D., Goldkorn A. (2018). Liquid biopsy in prostate cancer: Circulating tumor cells and beyond. Genitourinary Cancers.

[165] Meyer C.P., Pantel K., Tennstedt P., Stroelin P., Schlomm T., Heinzer H., Riethdorf S., Steuber T. (2016). Limited prognostic value of preoperative circulating tumor cells for early biochemical recurrence in patients with localized prostate cancer. Urol. Oncol. Semin. Orig. Investig..

[166] Doyen J., Alix-Panabières C., Hofman P., Parks S.K., Chamorey E., Naman H., Hannoun-Lévi J.-M. (2011). Circulating tumor cells in prostate cancer: A potential surrogate marker of survival. Crit. Rev. Oncol..

[167] Pachmann K., Clement J.H., Schneider C.-P., Willen B., Camara O., Pachmann U., Höffken K. (2005). Standardized quantification of circulating peripheral tumor cells from lung and breast cancer. Clin. Chem. Lab. Med..

[168] Dagogo-Jack I., Shaw A.T. (2018). Tumour heterogeneity and resistance to cancer therapies. Nat. Rev. Clin. Oncol..

[169] Brabletz T., Kalluri R., Nieto M.A., Weinberg R.A. (2018). EMT in cancer. Nat. Rev. Cancer.

[170] De Bono J.S., Scher H.I., Montgomery R.B., Parker C., Miller M.C., Tissing H., Doyle G.V., Terstappen L., Pienta K., Raghavan D. (2008). Circulating Tumor Cells Predict Survival Benefit from Treatment in Metastatic Castration-Resistant Prostate Cancer. Clin. Cancer Res..

[171] Greene S.B., Dago A.E., Leitz L.J., Wang Y., Lee J., Werner S.L., Gendreau S., Patel P., Jia S., Zhang L. (2016). Chromosomal Instability Estimation Based on Next Generation Sequencing and Single Cell Genome Wide Copy Number Variation Analysis. PLoS ONE.

[172] Stenzl A., Maas M., Hegemann M., Rausch S., Bedke J., Todenhöfer T. (2019). Circulating tumor cells and their role in prostate cancer. Asian J. Androl..

[173] Zhang T., Boominathan R., Foulk B., Rao C., Kemeny G., Strickler J.H., Abbruzzese J.L., Harrison M.R., Hsu D.S., Healy P. (2016). Development of a Novel c-MET-Based CTC Detection Platform. Mol. Cancer Res..

[174] Danila D.C., Anand A., Sung C.C., Heller G., Leversha M.A., Cao L., Lilja H., Molina A., Sawyers C.L., Fleisher M. (2011). TMPRSS2-ERG Status in Circulating Tumor Cells as a Predictive Biomarker of Sensitivity in Castration-Resistant Prostate Cancer Patients Treated with Abiraterone Acetate. Eur. Urol..

[175] Todenhöfer T., Azad A., Stewart C., Gao J., Eigl B., Gleave M., Joshua A., Black P.C., Chi K.N. (2016). AR-V7 Transcripts in Whole Blood RNA of Patients with Metastatic Castration Resistant Prostate Cancer Correlate with Response to Abiraterone Acetate. J. Urol..

[176] Antonarakis E.S., Lu C., Wang H., Luber B., Nakazawa M., Roeser J.C., Chen Y., Mohammad T.A., Chen Y., Fedor H.L. (2014). AR-V7 and Resistance to Enzalutamide and Abiraterone in Prostate Cancer. N. Engl. J. Med..

[177] Todenhöfer T., Hennenlotter J., Feyerabend S., Aufderklamm S., Mischinger J., Kühs U., Gerber V., Fetisch J., Schilling D., Hauch S. (2012). Preliminary experience on the use of the Adnatest® system for detection of circulating tumor cells in prostate cancer patients. Anticancer. Res..

[178] Qin X., Park S., Duffy S.P., Matthews K., Ang R.R., Todenhöfer T., Abdi H., Azad A., Bazov J., Chi K.N. (2015). Size and deformability based separation of circulating tumor cells from castrate resistant prostate cancer patients using resettable cell traps. Lab a Chip.

[179] De Giorgi V., Pinzani P., Salvianti F., Panelos J., Paglierani M., Janowska A., Grazzini M., Wechsler J., Orlando C., Santucci M. (2010). Application of a Filtration- and Isolation-by-Size Technique for the Detection of Circulating Tumor Cells in Cutaneous Melanoma. J. Investig. Dermatol..

[180] Todenhöfer T., Park E.S., Duffy S., Deng X., Jin C., Abdi H., Ma H., Black P.C. (2016). Microfluidic enrichment of circulating tumor cells in patients with clinically localized prostate cancer. Urol. Oncol. Semin. Orig. Investig..

[181] Stott S.L., Lee R.J., Nagrath S., Yu M., Miyamoto D.T., Ulkus L., Inserra E.J., Ulman M., Springer S., Nakamura Z. (2010). Isolation and characterization of circulating tumor cells from patients with localized and meta-static prostate cancer. Sci Transl Med..

[182] Danese E., Montagnana M., Lippi G. (2019). Circulating molecular biomarkers for screening or early diagnosis of colorectal cancer: Which is ready for prime time?. Ann Transl Med..

[183] Mukherji D., Jabbour M.N., Saroufim M., Temraz S., Nasr R., Charafeddine M., Assi R., Shamseddine A., Tawil A.N. (2016). Programmed Death-Ligand 1 Expression in Muscle-Invasive Bladder Cancer Cystectomy Specimens and Lymph Node Metastasis: A Reliable Treatment Selection Biomarker?. Clin. Genitourin. Cancer.

[184] Shin D.S., Zaretsky J.M., Escuin-Ordinas H., Garcia-Diaz A., Hu-Lieskovan S., Kalbasi A., Grasso C.S., Hugo W., Sand-oval S., Torrejon D.Y. (2017). Primary Resistance to PD-1 Blockade Mediated by JAK1/2 Mutations. Cancer Discov..

[185] Bidard F.-C., Peeters D., Fehm T., Nolé F., Gisbert-Criado R., Mavroudis D., Grisanti S., Generali D., Garcia-Saenz J.A., Stebbing J. (2014). Clinical validity of circulating tumour cells in patients with metastatic breast cancer: A pooled analysis of individual patient data. Lancet Oncol..

[186] Ghebeh H., Lehe C., Barhoush E., Al-Romaih K., Tulbah A., Al-Alwan M., Hendrayani S.-F., Manogaran P., Alaiya A., Al-Tweigeri T. (2010). Doxorubicin downregulates cell surface B7-H1 expression and upregulates its nuclear expression in breast cancer cells: Role of B7-H1 as an anti-apoptotic molecule. Breast Cancer Res..

[187] Ingebrigtsen V.A., Boye K., Tekle C., Nesland J.M., Flatmark K., Fodstad Ø. (2012). B7-H3 expression in colorectal cancer: Nuclear localization strongly predicts poor outcome in colon cancer. Int. J. Cancer.

[188] Rittmeyer A., Barlesi F., Waterkamp D., Park K., Ciardiello F., von Pawel J., Gadgeel S.M., Hida T., Kowalski D., Dols M.C. (2016). Atezolizumab versus docetaxel in patients with previously treated non-small-cell lung cancer (OAK): A phase 3, open-label, multicentre randomised controlled trial. Lancet.

[189] Ferrari C., De Panfilis G., Allegri L., Manara G.C. (1988). Detection of Cell Surface Antigens in Tissue Sections by Means of Pre-Embedding Immunogold Staining. Stain. Technol..

[190] Sholl L.M., Aisner D.L., Allen T.C., Beasley M.B., Cagle P.T., Capelozzi V.L., Dacic S., Hariri L.P., Kerr K.M., Lantuejoul S. (2016). Liquid Biopsy in Lung Cancer: A Perspective from Members of the Pulmonary Pathology Society. Arch. Pathol. Lab. Med..

[191] Olson B.M., Jankowska-Gan E., Becker J., Vignali D.A.A., Burlingham W.J., McNeel D.G. (2012). Human Prostate Tumor Antigen–Specific CD8+ Regulatory T Cells Are Inhibited by CTLA-4 or IL-35 Blockade. J. Immunol..

[192] McNeel D.G., Gardner T.A., Higano C.S., Kantoff P., Small E.J., Wener M.H., Sims R.B., Devries T., Sheikh N.A., Dreicer R. (2014). A Transient Increase in Eosinophils Is Associated with Prolonged Survival in Men with Metastatic Castration-Resistant Prostate Cancer Who Receive Sipuleucel-T. Cancer Immunol. Res..

[193] Di Meo A., Bartlett J., Cheng Y., Pasic M.D., Yousef G.M. (2017). Liquid biopsy: A step forward towards precision medicine in urologic malignancies. Mol Cancer..

[194] Antonarakis E.S., Lu C., Luber B., Wang H., Chen Y., Nakazawa M., Nadal R., Paller C.J., Denmeade S.R., Carducci M.A. (2015). Androgen Receptor Splice Variant 7 and Efficacy of Taxane Chemotherapy in Patients with Metastatic Castration-Resistant Prostate Cancer. JAMA Oncol..

[195] Markowski M.C., Silberstein J.L., Eshleman J.R., Eisenberger M.A., Luo J., Antonarakis E.S. (2017). Clinical Utility of CLIA-Grade AR-V7 Testing in Patients with Metastatic Castration-Resistant Prostate Cancer. JCO Precis. Oncol..

[196] Seitz A.K., Thoene S., Bietenbeck A., Nawroth R., Tauber R., Thalgott M., Schmid S., Secci R., Retz M., Gschwend J.E. (2017). AR-V7 in Peripheral Whole Blood of Patients with Castration-resistant Prostate Cancer: Association with Treatment-specific Outcome Under Abiraterone and Enzalutamide. Eur. Urol..

[197] Worroll D., Galletti G., Gjyrezi A., Nanus D.M., Tagawa S.T., Giannakakou P. (2019). Androgen receptor nuclear localization correlates with AR-V7 mRNA expression in circulating tumor cells (CTCs) from metastatic castration resistance prostate cancer patients. Phys. Biol..

[198] Panigrahi G.K., Deep G. (2017). Exosomes-based biomarker discovery for diagnosis and prognosis of prostate cancer. Front Biosci.

[199] Xie F., Xu M., Lu J., Mao L., Wang S. (2019). The role of exosomal PD-L1 in tumor progression and immunotherapy. Mol. Cancer.

[200] Xiong W., Gao Y., Wei W., Zhang J. (2021). Extracellular and nuclear PD-L1 in modulating cancer immunotherapy. Trends Cancer.

[201] Gong B., Kiyotani K., Sakata S., Nagano S., Kumehara S., Baba S., Besse B., Yanagitani N., Friboulet L., Nishio M. (2019). Secreted PD-L1 variants mediate resistance to PD-L1 blockade therapy in non–small cell lung cancer. J. Exp. Med..

[202] Poggio M., Hu T., Pai C.-C., Chu B., Belair C.D., Chang A., Montabana E., Lang U.E., Fu Q., Fong L. (2019). Suppression of exosomal PD-L1 induces systemic anti-tumor immunity and memory. Cell.

[203] Young C., Horton R. (2005). Putting clinical trials into context. Lancet.

[204] Oxman A.D., Cook D.J., Guyatt G.H., Bass E., Brill-Edwards P., Browman G., Detsky A., Farkouh M., Gerstein H., Haines T. (1994). Users’ Guides to the Medical Literature. JAMA.

[205] Swingler G.H., Volmink J., A Ioannidis J.P. (2003). Number of published systematic reviews and global burden of disease: Database analysis. BMJ.

[206] Moher D., Liberati A., Tetzlaff J., Altman D.G. (2009). PRISMA Group. Preferred reporting items for systematic reviews and me-ta-analyses: The PRISMA statement. Ann. Intern Med..

[207] Liberati A., Altman D.G., Tetzlaff J., Mulrow C., Gøtzsche P.C., Ioannidis J.P.A., Clarke M., Devereaux P.J., Kleijnen J., Moher D. (2009). The PRISMA statement for reporting systematic reviews and meta-analyses of studies that evaluate healthcare in-terventions: Explanation and elaboration. BMJ.

[208] Palicelli A., Giaccherini L., Zanelli M., Bonasoni M., Gelli M., Bisagni A., Zanetti E., De Marco L., Torricelli F., Manzotti G. (2021). How Can We Treat Vulvar Carcinoma in Pregnancy? A Systematic Review of the Literature. Cancers.

[209] Zanelli M., Sanguedolce F., Zizzo M., Palicelli A., Bassi M.C., Santandrea G., Martino G., Soriano A., Caprera C., Corsi M. (2021). Primary effusion lymphoma occurring in the setting of transplanted patients: A systematic review of a rare, life-threatening post-transplantation occurrence. BMC Cancer.

[210] Sanguedolce F., Zanelli M., Zizzo M., Bisagni A., Soriano A., Cocco G., Palicelli A., Santandrea G., Caprera C., Corsi M. (2021). Primary Pulmonary B-Cell Lymphoma: A Review and Update. Cancers.

[211] Page M.J., E McKenzie J., Bossuyt P.M., Boutron I., Hoffmann T.C., Mulrow C.D., Shamseer L., Tetzlaff J.M., Moher D. (2021). Updating guidance for reporting systematic reviews: Development of the PRISMA 2020 statement. J. Clin. Epidemiol..

[212] Bonasoni M.P., Palicelli A., Dalla Dea G., Comitini G., Pazzola G., Russello G., Bertoldi G., Bardaro M., Zuelli C., Carretto E. (2021). Kingella kingae Intrauterine Infection: An Unusual Cause of Chorioamnionitis and Miscarriage in a Patient with Undif-ferentiated Connective Tissue Disease. Diagnostics.

[213] Sanguedolce F., Calò B., Mancini V., Zanelli M., Palicelli A., Zizzo M., Ascani S., Carrieri G., Cormio L. (2021). Non-Muscle Invasive Bladder Cancer with Variant Histology: Biological Features and Clinical Implications. Oncology.

[214] Page M.J., McKenzie J.E., Bossuyt P.M., Boutron I., Hoffmann T.C., Mulrow C.D., Shamseer L., Tetzlaff J.M., Akl E.A., Brennan S.E. (2021). The PRISMA 2020 statement: An updated guideline for reporting systematic reviews. BMJ.

[215] Wing-Cheuk Wong R., Palicelli A., Hoang L., Singh N. (2020). Interpretation of p16, p53 and mismatch repair protein immuno-histochemistry in gynaecological neoplasia. Diagn. Histopathol..

[216] Liu L., Dong H., Jin X., Brooke-Wavell K. (2021). Tackling Dementia: A Systematic Review of Interventions Based on Physical Ac-tivity. J. Geriatr. Phys. Ther..

[217] Olivadese R., Ramponi A., Boldorini R., Dalla Dea G., Palicelli A. (2021). Mitotically Active Cellular Fibroma of the Ovary Recurring After the Longest Interval of Time (16 yr): A Challenging Case with Systematic Literature Review. Int. J. Gynecol. Pathol..

[218] Zanelli M., Ricci S., Zizzo M., Sanguedolce F., De Giorgi F., Palicelli A., Martino G., Ascani S. (2021). Systemic Mastocytosis As-sociated with “Smoldering” Multiple Myeloma. Diagnostics.

[219] Aleksandra C.Z., Iris H., Maarten J.V.L., Jean-Paul P.M.V., Michel M.P.J.R., Clark J.Z. (2021). Systematic Review on the Mid-Term Outcomes of Elective Endovascular Aneurysm Sealing in Comparison to Endovascular Aneurysm Repair. J. Endovasc. Ther..

[220] Bonasoni M.P., Palicelli A., Dea G.D., Comitini G., Nardini P., Vizzini L., Russello G., Bardaro M., Carretto E. (2021). *Klebsiella pneumoniae* Chorioamnionitis: An Underrecognized Cause of Preterm Premature Rupture of Membranes in the Second Trimester. Microorganisms.

[221] Palicelli A. (2020). What do we know about the cytological features of pure intraductal carcinomas of the salivary glands?. Cytopathology.

[222] Storoni S., Treurniet S., Micha D., Celli M., Bugiani M., van den Aardweg J.G., Eekhoff E.M.W. (2021). Pathophysiology of res-piratory failure in patients with osteogenesis imperfecta: A systematic review. Ann. Med..

[223] Zhong J., Slevin F., Scarsbrook A.F., Serra M., Choudhury A., Hoskin P.J., Brown S., Henry A.M. (2021). Salvage Reirradiation Options for Locally Recurrent Prostate Cancer: A Systematic Review. Front. Oncol..

[224] Palicelli A., Barbieri P., Mariani N., Re P., Galla S., Sorrentino R., Locatelli F., Salfi N., Valente G. (2018). Unicystic high-grade intraductal carcinoma of the parotid gland: Cytological and histological description with clinic-pathologic review of the liter-ature. APMIS.

[225] Porreca A., Colicchia M., Tafuri A., D’Agostino D., Busetto G.M., Crestani A., Odorizzi K., Amigoni N., Rizzetto R., Gozzo A. (2021). Perioperative Outcomes of Holmium Laser Enucleation of the Prostate: A Systematic Review. Urol. Int..

[226] Anderson E.M., Kim S., Sandler H.M., Kamrava M. (2021). High-dose-rate fractionated brachytherapy monotherapy for localized prostate cancer: A systematic review and meta-analysis. J. Contemp. Brachyther..

[227] Kočo L., Weekenstroo H.H.A., Lambregts D.M.J., Sedelaar J.P.M., Prokop M., Fütterer J.J., Mann R.M. (2021). The Effects of Multidisciplinary Team Meetings on Clinical Practice for Colorectal, Lung, Prostate and Breast Cancer: A Systematic Review. Cancers.

[228] Ardighieri L., Palicelli A., Ferrari F., Bugatti M., Drera E., Sartori E., Odicino F. (2020). Endometrial Carcinomas with Intesti-nal-Type Metaplasia/Differentiation: Does Mismatch Repair System Defects Matter? Case Report and Systematic Review of the Literature. J. Clin. Med..

[229] D’Agostino C., Surico D., Monga G., Palicelli A. (2019). Pregnancy-related decidualization of subcutaneous endometriosis occurring in a post-caesarean section scar: Case study and review of the literature. Pathol. Res. Pract..

[230] Palicelli A., Neri P., Marchioro G., De Angelis P., Bondonno G., Ramponi A. (2018). Paratesticular seminoma: Echographic features and histological diagnosis with review of the literature. APMIS.

[231] Disanto M.G., Mercalli F., Palicelli A., Arnulfo A., Boldorini R. (2017). A unique case of bilateral ovarian splenosis and review of the literature. APMIS.

[232] Palicelli A., Disanto M.G., Panzarasa G., Veggiani C., Galizia G., Cin S.D., Gruppioni E., Boldorini R. (2016). Orbital meningeal melanocytoma: Histological, immunohistochemical and molecular characterization of a case and review of the literature. Pathol. Res. Pract..

[233] Mandato V.D., Mastrofilippo V., Palicelli A., Silvotti M., Serra S., Giaccherini L., Aguzzoli L. (2021). Solitary vulvar metastasis from early-stage endometrial cancer: Case report and literature review. Medicine.

[234] Zanelli M., Smith M., Zizzo M., Carloni A., Valli R., De Marco L., Foroni M., Palicelli A., Martino G., Ascani S. (2019). A tricky and rare cause of pulmonary eosinophilia: Myeloid/lymphoid neoplasm with eosinophilia and rearrangement of PDGFRA. BMC Pulm. Med..

[235] Palicelli A., Boldorini R., Campisi P., Disanto M.G., Gatti L., Portigliotti L., Tosoni A., Rivasi F. (2016). Tungiasis in Italy: An imported case of Tunga penetrans and review of the literature. Pathol. Res. Pract..

[236] Ambrosetti F., Palicelli A., Bulfamante G., Rivasi F. (2013). Langer Mesomelic Dysplasia in Early Fetuses: Two Cases and a Literature Review. Fetal Pediatr. Pathol..

[237] Ardighieri L., Palicelli A., Ferrari F., Ragnoli M., Ghini I., Bugatti M., Bercich L., Sartori E., Odicino F.E. (2021). Risk Assessment in Solitary Fibrous Tumor of the Uterine Corpus: Report of a Case and Systematic Review of the Literature. Int. J. Surg. Pathol..

[238] Palicelli A. (2019). Intraductal carcinomas of the salivary glands: Systematic review and classification of 93 published cases. APMIS.

[239] Yao H.H.-I., Hoe V., Shamout S., Sengupta S., O’Connell H.E., Carlson K.V., Baverstock R.J. (2021). Impact of radiotherapy for localized prostate cancer on bladder function as demonstrated on urodynamics study: A systematic review. Can. Urol. Assoc. J..

[240] Zanelli M., Pizzi M., Sanguedolce F., Zizzo M., Palicelli A., Soriano A., Bisagni A., Martino G., Caprera C., Moretti M. (2021). Gastrointestinal Manifestations in Systemic Mastocytosis: The Need of a Multidisciplinary Approach. Cancers.

[241] Donegani E., Ambassa J.C., Mvondo C., Giamberti A., Ramponi A., Palicelli A., Chelo D. (2013). Linfoma di Burkitt cardiaco primitivo in un giovane ragazzo africano [Primary cardiac Burkitt lymphoma in an African child]. G. Ital. Cardiol..

[242] Bonasoni M.P., Comitini G., Barbieri V., Palicelli A., Salfi N., Pilu G. (2021). Fetal Presentation of Mediastinal Immature Teratoma: Ultrasound, Autopsy and Cytogenetic Findings. Diagnostics.

[243] Zanelli M., Sanguedolce F., Palicelli A., Zizzo M., Martino G., Caprera C., Fragliasso V., Soriano A., Valle L., Ricci S. (2021). EBV-Driven Lymphoproliferative Disorders and Lymphomas of the Gastrointestinal Tract: A Spectrum of Entities with a Common Denominator (Part 1). Cancers.

[244] Zanelli M., Sanguedolce F., Palicelli A., Zizzo M., Martino G., Caprera C., Fragliasso V., Soriano A., Valle L., Ricci S. (2021). EBV-Driven Lymphoproliferative Disorders and Lymphomas of the Gastrointestinal Tract: A Spectrum of Entities with a Common Denominator (Part 2). Cancers.

[245] Palicelli A., Bonacini M., Croci S., Magi-Galluzzi C., Cañete-Portillo S., Chaux A., Bisagni A., Zanetti E., De Biase D., Melli B. (2021). What Do We Have to Know about PD-L1 Expression in Prostate Cancer? A Systematic Literature Review. Part 1: Focus on Immunohistochemical Results with Discussion of Pre-Analytical and Interpretation Variables. Cells.

[246] Palicelli A., Bonacini M., Croci S., Magi-Galluzzi C., Cañete-Portillo S., Chaux A., Bisagni A., Zanetti E., De Biase D., Melli B. (2021). What do we have to know about PD-L1 expression in prostate cancer? A systematic literature review. Part 2: Clinic-pathologic correlations. Cells.

[247] Palicelli A., Croci S., Bisagni A., Zanetti E., De Biase D., Melli B., Sanguedolce F., Ragazzi M., Zanelli M., Chaux A. (2021). What Do We Have to Know about PD-L1 Expression in Prostate Cancer? A Systematic Literature Review. Part 3: PD-L1, Intracellular Signaling Pathways and Tumor Microenvironment. Int. J. Mol. Sci..

[248] Palicelli A., Croci S., Bisagni A., Zanetti E., De Biase D., Melli B., Sanguedolce F., Ragazzi M., Zanelli M., Chaux A. (2021). What Do We Have to Know about PD-L1 Expression in Prostate Cancer? A Systematic Literature Review. Part 4: Experimental Treatments in Pre-Clinical Studies (Cell Lines and Mouse Models). Int. J. Mol. Sci..

[249] Palicelli A., Croci S., Bisagni A., Zanetti E., De Biase D., Melli B., Sanguedolce F., Ragazzi M., Zanelli M., Chaux A. (2021). What Do We Have to Know about PD-L1 Expression in Prostate Cancer? A Systematic Literature Review. Part 5: Epigenetic Regulation of PD-L1. Int. J. Mol. Sci..

